# Proteomic response of hybrid wild rice to cold stress at the seedling stage

**DOI:** 10.1371/journal.pone.0198675

**Published:** 2018-06-07

**Authors:** Jinzi Wang, Jun Wang, Xin Wang, Rongbai Li, Baoshan Chen

**Affiliations:** 1 State Key Laboratory for Conservation and Utilization of Subtropical Agro-bioresources, Guangxi University, Nanning, China; 2 College of Agriculture, Guangxi University, Nanning, China; 3 College of Life Science and Technology, Guangxi University, Nanning, China; Louisiana State University, UNITED STATES

## Abstract

Low temperature at the seedling stage is a major damaging factor for rice production in southern China. To better understand the cold response of cultivated and wild rice, cold-sensitive cultivar 93–11 (*Oryza sativa* L. ssp. *Indica*) and cold-resistant hybrid wild rice DC907 with a 93–11 genetic background were used for a quantitative proteomic analysis with tandem mass tags (TMT) in parallel. Rice seedlings grown for four weeks at a normal temperature (25°C) were treated at 8–10°C for 24, 72 and 120 h. The number of differentially expressed proteins increased gradually over time in the cold-exposed rice in comparison with the untreated rice. A total of 366 unique proteins involved in ATP synthesis, photosystem, reactive oxygen species, stress response, cell growth and integrity were identified as responding to cold stress in DC907. While both DC907 and 93–11 underwent similar alterations in proteomic profiles in response to cold stress, DC907 responded in a prompter manner in terms of expressing cold-responding proteins, maintained a higher level of photosynthesis to power the cells, and possessed a stable and higher level of DIR proteins to prevent the plant from obtaining irreversible cell structure damage. The observations made in this study may lay a new foundation for further investigation of cold sensitivity or tolerance mechanisms in rice.

## Introduction

Low temperature is a major environmental stress affecting plant growth. Chilling stress causes water reduction and osmotic changes in the cellular milieu and suppresses the activities of cellular macromolecules, resulting in reduced growth and extensive losses in agricultural production [1]. Rice (*Oryza sativa*), a monocot plant and widely grown as food crop in tropical and subtropical areas, is particularly sensitive to cold stress at the seedling and flowering stages [2, 3]. Molecular genetic studies have already identified components of cold tolerance, such as *CTB4a*, which confers cold resistance by mediating ATP supply [4], and the *WRKY* gene superfamily in rice [5]. *COLD1*, as one of the best-characterized rice genes, is considered as a regulator of G-protein signaling (RGS) that regulates Ca^2+^ signaling in cells and confers chilling tolerance in rice [2, 6]. The dehydrin gene *OsDhn1* has been identified as being highly expressed in developing seeds under low temperatures and protects rice floral organs against abiotic stress [7]. *qCTS-9*, found in hybrid rice under different cold environments, was confirmed to be a functional gene associated with cold tolerance at rice seedling stage [8]. In a genome-wide association mapping of cold tolerance in cultivated rice from rice diversity panel 1 (RDP1), 87 cold tolerance-related quantitative trait loci (QTLs) with significant enrichment for genes related to lipid metabolism, response to stress and oxygen binding were identified [9, 10].

Recently, proteomic technologies have been used to monitor and characterize protein profiles in rice [11]. For example, two-dimensional gel electrophoresis (2-DE) and isobaric tags labelling approaches were used to monitor the proteomic response of rice to the cold treatment and proteins involved in energy metabolism, transport, photosynthesis, precursor metabolites generation, histones and vitamin B biosynthesis, which were found to be differentially expressed by cold stress [12–14]. However, to date, only a limited number of proteins in the cold-response pathway have been identified.

Early season rice in South China generally suffers from cold weather characterized by a temperature drop to approximately 10°C or lower that results in seedling rot one in every three years in mid- to late March [15]. However, wild rice (*Oryza rufipogon* Griff.) in the same region survives the cold stress. Efforts to introduce the cold tolerance trait of wild rice into cultivar rice have been carried out by crossing cultivar rice with wild rice. To better understand the cold resistance mechanism of wild rice, a cold-tolerant hybrid wild rice DC907 with cultivar 93–11 genetic background and cold-sensitive 93–11 were investigated in parallel in this study by a comparative proteomics approach. Our results show that cold-tolerant DC907 was different from cold-sensitive 93–11 in its protein expression pattern. While a small portion of the differentially expressed proteins match those previously reported, a large proportion of cold stress-induced proteins were reported for the first time.

## Materials and methods

### Rice growth and cold treatment conditions

Seeds of the *indica* rice cultivar 93–11 and hybrid wild rice DC907, derived from crossing of Guangxi wild rice (*Oryza rufipogon* Griff.) with 93–11 and sequential back cross with 93–11 for 4 rounds, were germinated in soil and grown in a phytotron with a 12-h day/night cycle, at 25°C in the day and 18°C at night. Seedlings at the four-leaf stage were subject to cold treatment at 10°C in the day and 8°C at night to simulate natural cold conditions for varied time durations. Seedlings were separated into groups for varied cold treatment conditions. Each group contained a total of 100 individual plants. After cold treatment for a fixed amount of time, plants were then transferred to an environment of 25°C in the day and 18°C at night for 5 days for survival rate determination, defined as the ratio of surviving plants to total plants. The light intensity was set at 30000 lux.

### Preparation of protein samples for TMT analysis

Whole rice seedlings were ground into powder with liquid nitrogen and then five volumes of pre-cold acetone containing 10% trichloroacetic acid (TCA) and 0.07% β-mercaptoethanol was added. The mixture was kept at -20°C overnight and centrifuged at 18,000 g for 30 min. The crude protein pellet was washed with pre-cold acetone containing 0.07% β-mercaptoethanol three times by centrifugation at 18,000 g. After vacuum drying, lysis buffer (7 M urea, 2% 3-((3-cholamidopropyl) dimethylammonio)-1-propanesulfonate (CHAPS), 40 mM Tris, 1 mM phenylmethanesulfonyl fluoride (PMSF)) was added to dissolve the protein pellet that was then centrifuged at 18,000 g for 30 min to remove debris. Five volumes of pre-chilled acetone was added to the supernatant and kept at -20°C overnight. The mixture was centrifuged at 18,000 g for 30 min, and the pellet was dissolved in triethylamine borane (TEAB, 100 mM). The protein concentration was determined using the Bradford method [16].

An amount of 100 μg of protein was mixed with 5 μl of 200 mM tris (2-carboxyethyl) phosphine (TCEP) and incubated at 55°C for 60 min. Then, 5 μl of 375 mM iodoacetamide was added and incubated for another 30 min in the dark. Six volumes of pre-cold acetone was added to precipitate proteins overnight. The protein mixture was centrifuged at 18,000 g for 30 min, and the pellet was dried at room temperature and dissolved in 100 μl of triethylamonium bicarbonat (TEAB). Trypsin solution (2.5 μg/100 μg protein) was used to digest protein samples at 37°C overnight. The peptides were labeled with 41 μl of the TMTsixplex label reagent set (Thermo Fisher Scientific, CAT 90061) and incubated for 60 min at room temperature. Then, 8 μl of 5% hydroxylamine was used to quench the labeling reaction for 15 min, and the labeled peptides were stored at -80°C.

### Strong cation exchange chromatography

A PolyLC polysulfoethyl aspartamide column (100 mm X 2.1 mm, 5 μm, 300Å pore size) was used on a Waters high-performance liquid chromatography system (HPLC, Waters, series 2695) for off-line strong-cation exchange (SCX) chromatography fractionation. A 40 min gradient elution of 100% solvent A (10 mM monopotassium phosphate, 15% acetonitrile) to 100% solvent B (500 mM potassium chloride in solvent A) at 200 μl/min flow rate was performed. The SCX elution was monitored under a Waters 2998 PDA detector module (220 nm) and collected into 25 fractions for further mass spectrometry (MS) analysis.

### Nanoflow LC-MS/MS analysis and data processing

The SCX fractions were loaded onto the trap column (nanoViper C18, 75 μm X 2 cm) and then eluted using capillary analytical column (nanoViper C18, 50 μm X 15 cm) at a 300 nl/min flow rate using Easy-nLC 1000 nanoflow liquid chromatography system (Thermo Fisher Scientific). The linear gradient for peptide elution was from 95% solvent A (0.1% formic acid) and 5% solvent B (0.1% formic acid, 98% acetonitrile) to 40% solvent B for a 60 min program. The peptides from the untreated control and cold treated samples were labeled, mixed and fractionated by SCX chromatography and then analyzed using LTQ-Orbitrap Elite hybrid mass spectrometer system. Three biological replicates for each sample were performed.

The scan range of mass spectrometric analysis was set at 350–1800 m/z in a data-dependent mode. The survey scan was set at 400 m/z with a mass resolution of 60,000. Tandem mass spectrometry (MS/MS or MS2) was preceded with ten of the most intense precursor ions in the collision-induced dissociation (CID) mode with 35% normalized collision energy. MS2 spectrum was acquired in the ion trap analyzer at normal speed. The software Proteome Discoverer 1.3 was used to search the mass spectrometric data against rice genome database v7.0 (http://rice.plantbiology.msu.edu/). Search parameters were set as a standard method: 2 missed cleavages using trypsin as endoprotease, lysine residues as fixed modification, peptide N-termini as variable modification, 10 ppm precursor ion mass tolerance, 0.8 Da fragment mass tolerance, and 1% maximum false discovery rate (FDR). The identified proteins were filtered with high peptide confidence. Proteins with a 1.5-fold change (p<0.05) were considered to be differentially expressed.

### Western blot analysis

A Western blot analysis was carried out according to a previous study [17]. Samples of 40 μg of total protein were loaded on sodium dodecyl sulfate polyacrylamide gel electrophoresis (SDS-PAGE) gel and transferred to polyvinylidene fluoride (PVDF) membranes using a semi-dry transfer unit after electrophoretic separation. Specific antibodies against ribulose bisphosphate carboxylase oxygenase (Rubisco) and glyceraldehyde-3-phosphate dehydrogenase (GAPDH) were purchased from Abcam (California, USA) and used to detect and verify the protein expression level in different samples by enhanced chemiluminescence (SuperSignal West Pico Substrate, Thermo Scientific).

### Bioinformatics

Identified differentially expressed proteins were classified according to Gene Ontology (GO) and KEGG. The protein information from the Database of Rice Genome Annotation Project [18] was converted to corresponding access numbers of the UniProt protein database and classified using QuickGo online annotation tool (http://www.ebi.ac.uk/QuickGO/GMultiTerm) [19]. The protein network of differentially expressed proteins was performed using agriGO v2.0 [20].

## Results and discussion

### Cold-response proteins and their time course

**Fig 1:**
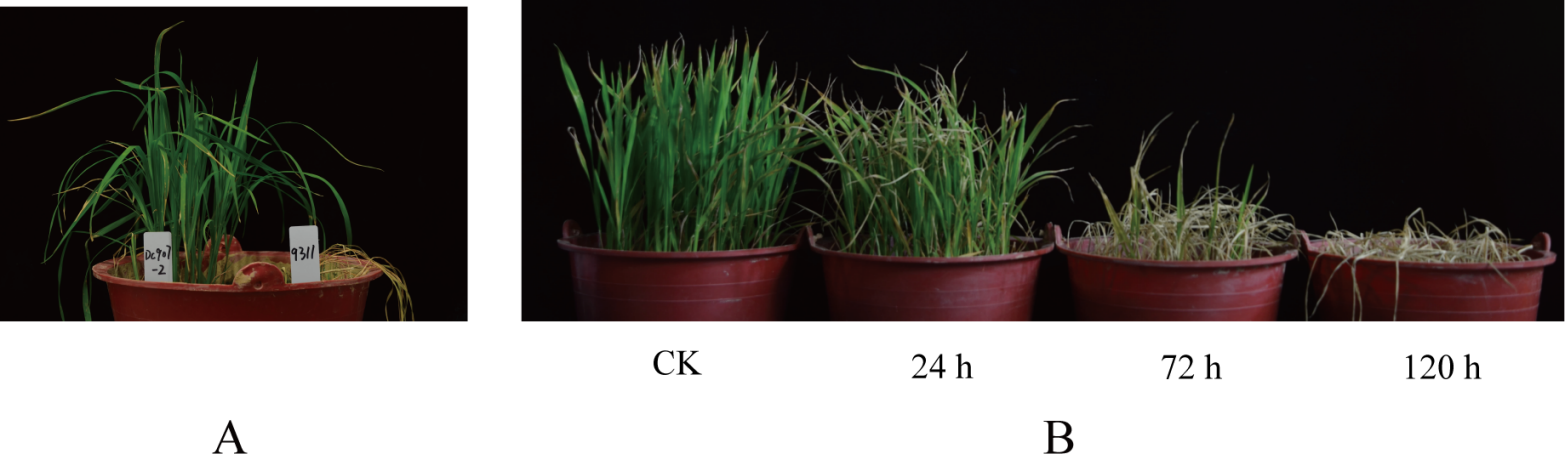
Phenotypes of low-temperature treated cultivar rice 93–11 after 5 days of recovery. Rice seedlings were cultured in a phytotron under light and temperature controls (the day/night cycle: 12 h with 25°C and 12 h with 18°C). The samples labeled as 24 h, 72 h and 120 h were treated under corresponding artificial low temperatures and recovered for 5 days under normal culture conditions. A, comparative phenotypes of 120 h low-temperature treated hybrid wild and cultivar rice after 5 days recovery. B, cultivar rice phenotypes of different low-temperature treatment times after 5 days of recovery.

**Fig 2:**
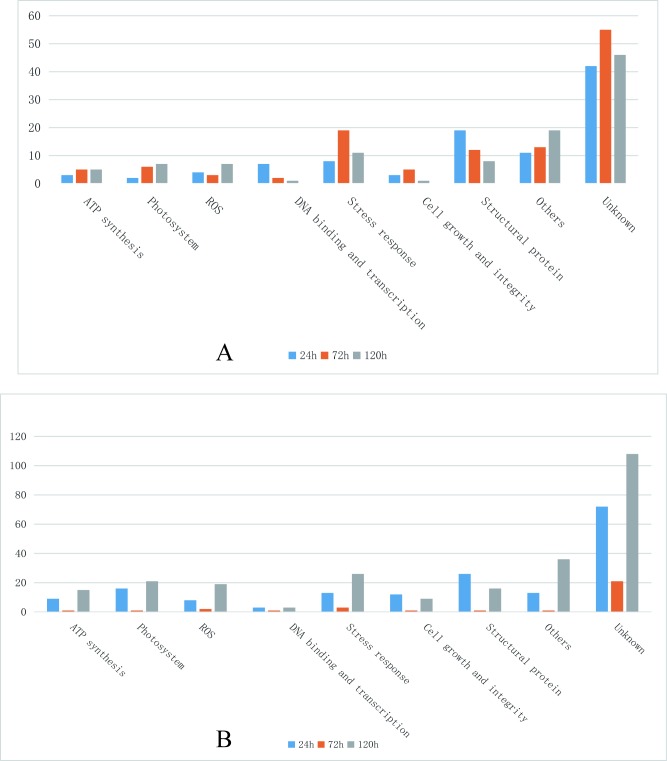
Classification of differentially expressed proteins. A, numbers of differentially expressed proteins between hybrid wild rice DC-907 and cultivar rice 93–11 at the same cold treatment time points. B, numbers of differentially expressed proteins in hybrid wild rice DC-907 at different cold treatment time points.

**Fig 3:**
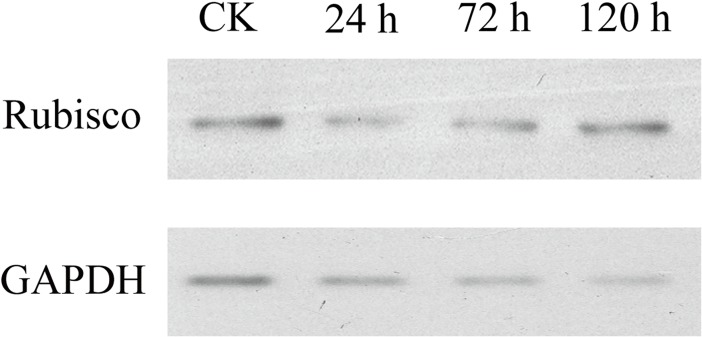
Western blot analysis of cold-treated hybrid wild rice. An amount of 40 μg of total protein from different samples was used for the Western blot analysis by the enhanced chemiluminescence (ECL) method. The specific antibodies against rubisco (1:1000) and GAPDH (1:1000) were used to detect the corresponding protein expressions. The change trends of these two proteins was consistent with the observations from TMT labeling.

Cold stress that is harmful to the seedlings of early season rice in South China (mid- to late March) is typically at approximately 10°C [15]. Thus, temperatures of 8–10°C were selected for cold treatment in this study. Based on our previous observations that 93–11 primarily survived cold stress for 24 h but died completely after 120 h (survival rate was counted at day 5 after the stress was relieved) and wild rice survived at both conditions, we opted to use 24, 72, and 120 h as the times for proteomic analysis. As seen in Fig 1, the survival rates of 93–11 after cold stress for 24 h, 72 h, and 120 h were 90%, 20%, and 0%, respectively, while the survival rates of DC907 were 100% at all time points. A total of 1781 unique proteins were identified by TMT labeling from cold-stressed DC907 and 93–11 (S1 Table). By comparing protein abundance between the two accessions, 99 proteins (75 up- and 24 down-regulated) with changes greater than 1.5-fold were found after the first 24 h of cold treatment (Table 1). As the cold treatment time was extended, the number of differentially expressed proteins slightly increased: 120 (37 up- and 83 down-regulated) at 72 h (Table 2) and 105 (46 up- and 59 down-regulated) at 120 h (Table 3). These proteins fall into different functional groups with obvious time course characteristics; ATP synthesis, photosystems and other functional groups increased while DNA binding and transcription, cell growth and integrity, and structural protein decreased as the cold treatment time was extended (Fig 2A). The structural proteins, DNA binding proteins, and transcriptional factors were among the most differentially expressed at the time point of 24 h, and the most significant changes found at 72 h were the proteins involved in stress response. Accumulations of protein related to photosynthesis and ATP synthesis were affected more severely in 93–11 as the cold treatment proceeded. A total of 366 unique proteins from DC907 were found to significantly change in terms of accumulation under cold treatment for all three time points when using the untreated sample as a reference (S2 Table). More interestingly, we found that most of the differentially expressed proteins at 24 h returned to the level at time zero at 72 h, but changed to an even higher level as the cold treatment continued to 120 h in DC907 (Fig 2B). However, no such change was found in 93–11. According to these observations, we assume that self-regulation in the hybrid wild rice is important for cold resistance to ensure that the expression level of key proteins would not overload and threaten plant survival. Compared with previous studies of cold-response proteomics of cultivar rice, a proportion of proteins were identified that included rubisco and GAPDH whose expression level were both decreased under cold stress [12, 13]. As shown in Fig 3, the expression level of rubisco and GAPDH in DC907 detected by Western blotting also decreased, in good accordance with TMT quantification.

**Table 1:** List of differentially expressed proteins post cold treatment for 24 h.

Protein ID ^a^	Description	T1/C1^b^
**ATP synthesis**		
Q6ZG90	ATP synthase	1.73
P42862	Glucose-6-phosphate isomerase, cytosolic A	1.58
Q69WE3	NADH-ubiquinone oxidoreductase-related-like protein	1.53
**Photosystem**		
Q84M34	Cytochrome b-c1 complex subunit 7	1.67
Q10F16	Ferredoxin	0.65
**ROS**		
B9FSC8	Putative 12-oxophytodienoate reductase 11	0.66
Q6H759	Copper chaperone homolog CCH	0.54
Q75IS1	Peroxidase	0.42
P0C5D1	1-Cys peroxiredoxin B	0.39
**DNA binding and transcription**		
Q94HA1	Gibberellin stimulated transcript related protein 1	3.39
Q5Z7N3	HMG protein	2.85
Q7XQK2	HMG protein	1.98
Q6YTY3	PHD finger protein ALFIN-LIKE 9	1.61
Q84Q79	LIM-domain protein	1.58
Q0DKM4	U1 small nuclear ribonucleoprotein A	1.57
Q10GH8	KH domain containing protein, expressed	0.59
**Stress response**		
Q5QM60	Non-specific lipid-transfer protein	1.82
Q6K8D4	Peptidylprolyl isomerase	1.66
Q2QYL3	Non-specific lipid-transfer protein 3	1.62
Q0J4P2	Heat shock protein 81–1	1.60
A0A0P0WNP9	Non-specific lipid-transfer protein (Fragment)	1.58
Q75HZ0	putative late embryogenesis abundant protein, LEA14-A	0.66
Q0JMY8	Salt stress-induced protein	0.64
Q8S3P3	DUF26-like protein	0.47
**Cell growth and integrity**		
Q851Y9	Nascent polypeptide-associated complex subunit beta	2.28
Q8L4E7	SAP-like protein BP-73	1.55
Q0JEF5	Flowering-promoting factor 1-like protein 4	0.42
**Structural protein**		
P31674	40S ribosomal protein S15	4.77
Q8SAY0	50S ribosomal protein L18, chloroplastic	3.57
Q7XEQ3	40S ribosomal protein S17-4, putative, expressed	3.00
Q53QG2	40S ribosomal protein S25, putative, expressed	2.94
Q6YY64	60S ribosomal protein L6	2.90
Q7XUC9	Histone H4	2.89
Q0IQF7	40S ribosomal protein S16	2.76
Q762A6	60S ribosomal protein L22-2, putative, expressed	2.73
Q2QNF3	60S ribosomal protein L2	2.68
P49398	40S ribosomal protein S4	2.53
Q84M35	40S ribosomal protein S2, putative, expressed	2.38
Q6ZL42	Probable histone H2A.2	2.28
Q10L93	50S ribosomal protein L6, putative, expressed	2.28
Q9ZST1	30S ribosomal protein S17, chloroplastic	2.15
Q851P9	Histone-like protein	1.98
P12153	30S ribosomal protein S19, chloroplastic	1.93
A0A0P0WK98	Ribosomal protein L15 (Fragment)	1.73
Q9AV77	60S ribosomal protein L17	1.70
Q7XKE9	Clathrin light chain 1	1.57
**Others**		
Q688X1	Eukaryotic translation initiation factor 3 subunit D	2.01
Q5Z627	Elongation factor 1-gamma 3	1.87
Q10LV9	Eukaryotic translation initiation factor 2 beta subunit, putative, expressed	1.78
Q9AUW3	Eukaryotic translation initiation factor 5A	1.67
Q5SMX7	Translation machinery-associated protein 22	1.65
Q10HX5	Modifier of rudimentary protein, expressed	1.57
Q948T6	Lactoylglutathione lyase	1.54
Q0DJA0	Coatomer subunit delta-1	1.51
Q9XEA6	Cysteine synthase	0.66
Q0DYB1	Soluble inorganic pyrophosphatase	0.57
Q6ZJX8	33-kDa secretory protein	0.39
**Unknown**		
Q0DWC5	Os02g0821200 protein (Fragment)	4.59
A0A0P0VUA6	Os03g0200500 protein (Fragment)	3.17
Q6K1W6	Os09g0258600 protein	2.98
Q6ZLB8	Os07g0180900 protein	2.88
Q8SA35	Os01g0659200 protein	2.81
A0A0P0XW06	Os10g0465800 protein (Fragment)	2.52
Q0E032	Os02g0581100 protein	2.48
A0A0N7KGC5	Os02g0821800 protein	2.48
Q5TKP2	Os05g0541900 protein	2.44
Q6YZI5	Os08g0558900 protein	2.30
Q6ZIA1	Os08g0530200 protein	2.23
Q84ZP1	Os07g0208000 protein	2.11
A0A0P0VUL0	Os03g0210600 protein (Fragment)	2.05
Q8S7H8	Os03g0778100 protein	1.87
Q5Z9Z8	Os06g0319700 protein	1.84
Q6H7T1	Os02g0162500 protein	1.78
A0A0N7KSQ1	Os11g0250000 protein	1.76
Q9FP98	Os01g0626300 protein	1.70
Q2RBP5	Os11g0103900 protein	1.66
Q7XI22	Os07g0186400 protein	1.62
Q6KA00	Os02g0822600 protein	1.61
Q2QWN3	Os12g0189400 protein	1.60
Q5VRC9	Os01g0179300 protein	1.59
Q5JN45	Os01g0959000 protein	1.59
Q8H3M0	Os08g0428800 protein	1.55
Q5W6H1	Os05g0350500 protein	1.55
Q7XIE2	Os07g0164300 protein	1.54
Q0J0C4	Os09g0517000 protein	1.54
Q84M68	Os03g0856500 protein	1.53
Q0DFD6	Os05g0597100 protein	1.53
A0A0P0XA87	Os07g0673500 protein	1.51
Q6AVR1	Expressed protein	0.65
Q650Y5	Os09g0564000 protein	0.65
Q943W1	Os01g0501800 protein	0.64
Q7G649	Expressed protein	0.64
A0A0P0V7A3	Os01g0711400 protein (Fragment)	0.64
Q10N30	Os03g0284400 protein	0.63
Q67IZ7	Os09g0461800 protein	0.63
B9FCM4	Os04g0626400 protein	0.63
Q75T45	Os12g0555000 protein	0.61
Q5Z6B8	Os06g0530200 protein	0.56
A0A0P0XC80	Os08g0169300 protein	0.04

a: the protein ID come from UniProt databse.

b: T1 represents hybrid wild rice DC907; C1 represents cultivar rice 93–11.

**Table 2:** List of differentially expressed proteins post cold treatment for 72 h.

Protein ID^a^	Description	T2/C2^b^
**ATP synthesis**		
Q6K5G8	Glyceraldehyde-3-phosphate dehydrogenase 3, cytosolic	2.16
Q40677	Fructose-bisphosphate aldolase, chloroplastic	1.82
Q0JHF8	Fructose-1,6-bisphosphatase, cytosolic	1.60
Q69WE3	NADH-ubiquinone oxidoreductase-related-like protein	0.63
Q84PA4	ATP synthase B chain, chloroplast, putative, expressed	0.49
**Photosystem**		
Q5ZA98	Chlorophyll a-b binding protein, chloroplastic	2.15
P18566	Ribulose bisphosphate carboxylase small chain A, chloroplastic	1.68
Q10HD0	Chlorophyll a-b binding protein, chloroplastic	1.60
Q0JG75	Photosystem II reaction center PSB28 protein, chloroplastic	0.60
Q0DFC9	Plastocyanin, chloroplastic	0.55
Q0DI31	Cytochrome c	0.50
**ROS**		
P28757	Superoxide dismutase [Cu-Zn] 2	0.65
P37834	Peroxidase 1	0.57
Q75IS1	Peroxidase	0.53
**DNA binding and transcription**		
Q6YTY3	PHD finger protein ALFIN-LIKE 9	1.70
Q8RUI4	NAC transcription factor	0.54
**Stress response**		
Q6ZKC0	14-3-3-like protein GF14-C	1.56
Q53NM9	DnaK-type molecular chaperone hsp70-rice	1.56
Q5WMX0	Drought Induced Protein 3, DIP3	1.51
Q07078	Heat shock protein 81–3	1.51
Q6K3Y6	NOI protein	0.67
Q10M12	Ricin B-like lectin R40C1	0.66
Q2QYK8	Non-specific lipid-transfer protein	0.65
Q7Y139	Huntingtin interacting protein K, putative, expressed	0.64
Q6ZBZ2	Germin-like protein 8–14	0.64
Q2QQ99	Protein SPIRAL1-like 3	0.63
Q0IQK9	Non-specific lipid-transfer protein 1	0.61
A0A0P0WNP9	Non-specific lipid-transfer protein (Fragment)	0.60
Q656V1	Peptidylprolyl isomerase	0.58
P25778	Oryzain gamma chain	0.54
Q10KY5	10 kDa chaperonin, putative, expressed	0.52
Q7XJ39	Non-specific lipid-transfer protein 2A	0.52
Q5QM60	Non-specific lipid-transfer protein	0.50
Q8S3P3	DUF26-like protein	0.48
Q2QYL3	Non-specific lipid-transfer protein 3	0.28
**Cell growth and integrity**		
Q6ZH98	Peptidyl-prolyl cis-trans isomerase	0.66
Q5QLS1	Arabinogalactan protein-like	0.64
P35681	Translationally-controlled tumor protein homolog	0.64
Q942D4	BURP domain-containing protein 3	0.63
Q8LMR3	Nascent polypeptide-associated complex alpha subunit, putative, expressed	0.53
**Structural protein**		
Q10DV7	Actin-1	2.91
A0A0P0WK98	Ribosomal protein L15 (Fragment)	1.78
P0C440	50S ribosomal protein L14, chloroplastic	1.76
Q7XUC9	Histone H4	1.63
Q2R1J8	40S ribosomal protein S9, putative, expressed	1.57
P35687	40S ribosomal protein S21	0.65
Q6YY64	60S ribosomal protein L6	0.65
P40978	40S ribosomal protein S19	0.64
Q2QS71	Probable histone H2A.7	0.55
P12153	30S ribosomal protein S19, chloroplastic	0.52
Q10PV6	50S ribosomal protein L15, chloroplast, putative, expressed	0.45
Q10MS5	40S ribosomal protein S7, putative, expressed	0.38
**Others**		
A0A0P0VMA7	Carboxypeptidase (Fragment)	1.95
Q2QLY5	5-methyltetrahydropteroyltriglutamate—homocysteine methyltransferase 1	1.87
Q5Z627	Elongation factor 1-gamma 3	1.58
Q0DJ99	Coatomer subunit delta-2	1.58
Q9LGQ6	Acyl transferase 9	1.52
Q75I27	Cucumisin-like serine protease, putative, expressed	0.67
Q84P97	Mitochondrial outer membrane protein porin 5	0.66
Q6Z730	Eukaryotic translation initiation factor 3 subunit J	0.65
Q6Z6H0	4-hydroxy-4-methyl-2-oxoglutarate aldolase	0.63
Q84MN8	Bifunctional 3'-phosphoadenosine 5'-phosphosulfate synthethase, putative, expressed	0.63
Q7XCS3	Cys/Met metabolism PLP-dependent enzyme family protein, expressed	0.62
Q0DYB1	Soluble inorganic pyrophosphatase	0.57
Q9LGE6	Probable U6 snRNA-associated Sm-like protein LSm4	0.51
**Unknown**		
Q0E032	Os02g0581100 protein	2.77
Q8S7H8	Os03g0778100 protein	1.92
Q6F385	Expressed protein	1.92
A0A0P0W2S8	Os03g0704100 protein (Fragment)	1.78
A0A0N7KKC7	Os05g0218400 protein	1.77
Q6ERL4	Os09g0338400 protein	1.71
Q5Z6P4	Os06g0264800 protein	1.70
Q7XT44	OSJNBb0089K24.3 protein	1.63
Q0D6L9	Os07g0467200 protein	1.62
Q69NF7	Os09g0530000 protein	1.61
Q7XPV4	OSJNBa0088H09.2 protein	1.61
Q652L5	Os09g0567350 protein	1.60
Q6YS11	Os08g0282400 protein	1.59
Q8W3J0	Os03g0278000 protein	1.55
Q6ZFH9	Os08g0503200 protein	1.51
Q2QWN3	Os12g0189400 protein	1.51
B9FCM4	Os04g0626400 protein	0.67
Q8H3M0	Os08g0428800 protein	0.66
Q84SC3	Os08g0162800 protein	0.66
A0A0P0VTB1	Os03g0157600 protein	0.66
Q6ZI51	Os02g0595800 protein	0.65
Q8S1F2	Os01g0588000 protein	0.65
Q5JMG1	Os01g0763300 protein	0.64
B9ETE4	Os01g0175000 protein	0.64
C7J6Y0	Os09g0482780 protein	0.64
B7FAF1	Os03g0222600 protein	0.64
Q6YZI5	Os08g0558900 protein	0.63
Q6EQG6	Os09g0345500 protein	0.63
Q67IZ7	Os09g0461800 protein	0.63
Q6PL11	Os11g0456300 protein	0.63
Q0DWC5	Os02g0821200 protein (Fragment)	0.63
Q7XTL6	OSJNBa0070M12.12 protein	0.62
B7E914	Os04g0310500 protein	0.62
A0A0P0W4K0	Os03g0807700 protein (Fragment)	0.62
Q6Z0W5	Os02g0308400 protein	0.62
Q8H3S1	Os08g0321000 protein	0.61
Q7XJ15	Os09g0541700 protein	0.61
Q2QND9	Expressed protein	0.60
Q75LJ7	Os03g0836200 protein	0.60
Q0DGH0	Os05g0533100 protein (Fragment)	0.60
C7JA48	Os12g0478100 protein (Fragment)	0.60
Q5TKP2	Os05g0541900 protein	0.59
A0A0N7KJ67	Os04g0462900 protein (Fragment)	0.58
Q5Z645	Os06g0567200 protein	0.58
Q0E446	Os02g0137200 protein (Fragment)	0.57
Q6ZKI0	Os08g0139200 protein	0.56
A0A0P0Y253	Os11g0472000 protein (Fragment)	0.56
Q0IVE4	Os10g0576000 protein (Fragment)	0.56
Q0DK70	Os05g0188100 protein	0.55
A0A0P0XRR7	Os09g0568900 protein (Fragment)	0.52
Q2R176	Os11g0615200 protein	0.51
Q6EUQ5	Os02g0175800 protein	0.48
Q6K1W6	Os09g0258600 protein	0.43
Q5Z6B8	Os06g0530200 protein	0.40
Q6ZIA1	Os08g0530200 protein	0.37

a: the protein ID come from UniProt databse.

b: T2 represents hybrid wild rice DC907; C2 represents cultivar rice 93–11.

**Table 3:** List of differentially expressed proteins post cold treatment for 120 h.

Protein ID^a^	Description	T3/C3^b^
**ATP synthesis**		
Q6ETN3	Probable 4-coumarate—CoA ligase 3	1.63
Q7X8A1	Glyceraldehyde-3-phosphate dehydrogenase	0.66
B9FK36	Acetyl-CoA carboxylase 2	0.66
Q7F280	Isocitrate dehydrogenase [NADP]	0.60
Q0JHF8	Fructose-1,6-bisphosphatase, cytosolic	0.60
**Photosystem**		
Q10F16	Ferredoxin	0.65
Q53N83	Chlorophyll a-b binding protein, chloroplastic	0.65
Q10HD0	Chlorophyll a-b binding protein, chloroplastic	0.61
Q7XV11	Chlorophyll a-b binding protein, chloroplastic	0.60
Q6Z411	Chlorophyll a-b binding protein, chloroplastic	0.51
P18566	Ribulose bisphosphate carboxylase small chain A, chloroplastic	0.43
Q5ZA98	Chlorophyll a-b binding protein, chloroplastic	0.39
**ROS**		
Q8L3W2	Peroxidase	1.94
Q7F1U0	Peroxidase	1.82
Q7XKD0	Thioredoxin X, chloroplastic	1.58
Q0D3N0	Peroxidase 2	1.53
Q6EUS1	Peroxidase	1.52
Q8L5K0	Ferritin	0.65
Q9SDD6	Peroxiredoxin-2F, mitochondrial	0.54
**DNA binding and transcription**		
Q7XQK2	HMG protein	0.64
**Stress response**		
Q7G2B5	Nonspecific lipid-transfer protein 2, putative, expressed	3.83
Q6ZBZ2	Germin-like protein 8–14	2.88
Q7XJ39	Non-specific lipid-transfer protein 2A	1.96
Q2QYK8	Non-specific lipid-transfer protein	1.92
Q2QYL0	Non-specific lipid-transfer protein	1.75
Q5QM60	Non-specific lipid-transfer protein	1.72
A0A0P0WNP9	Non-specific lipid-transfer protein (Fragment)	1.71
Q7Y139	Huntingtin interacting protein K, putative, expressed	1.59
Q9ASH1	Membrane-associated salt-inducible protein-like	0.60
Q07078	Heat shock protein 81–3	0.56
Q2R2W2	14-3-3-like protein GF14-D	0.38
**Cell growth and integrity**		
Q942D4	BURP domain-containing protein 3	1.50
**Structural protein**		
Q75HX0	Actin	1.63
Q84M35	40S ribosomal protein S2, putative, expressed	0.64
Q7XKE9	Clathrin light chain 1	0.64
O22386	50S ribosomal protein L12, chloroplastic	0.61
Q9AV77	60S ribosomal protein L17	0.60
Q75G91	40S ribosomal protein S3, putative, expressed	0.55
P49210	60S ribosomal protein L9	0.51
Q6K5R5	40S ribosomal protein S27	0.38
**Others**		
Q10R17	Adenylosuccinate synthetase 1, chloroplastic	2.42
A0A0P0V9F2	Cysteine proteinase inhibitor (Fragment)	2.28
Q8LMR0	Phosphoserine aminotransferase	1.69
Q5N8G1	2-C-methyl-D-erythritol 4-phosphate cytidylyltransferase, chloroplastic	1.64
Q6ERX1	Probable cinnamyl alcohol dehydrogenase 8A	1.56
Q69U53	MAP3K-like protein	1.54
Q0DYB1	Soluble inorganic pyrophosphatase	1.54
Q2QLY4	5-methyltetrahydropteroyltriglutamate—homocysteine methyltransferase 2	0.67
Q2QQ48	Eukaryotic translation initiation factor 5A	0.66
Q6ESI7	Tripeptidyl-peptidase 2	0.63
Q0DJ99	Coatomer subunit delta-2	0.63
Q5N7L5	Met-tRNAi formyl transferase-like	0.63
Q10PB3	Translocase of chloroplast	0.62
A2ZVI7	Calcium-dependent protein kinase 1	0.60
Q7F2G3	Carbonic anhydrase	0.56
P0C579	Cysteine proteinase inhibitor 10	0.55
Q5Z627	Elongation factor 1-gamma 3	0.53
Q6H7I9	ATP-dependent Clp protease proteolytic subunit	0.43
Q0IPE5	2-dehydro-3-deoxyphosphooctonate aldolase,	0.43
**Unknown**		
B7E914	Os04g0310500 protein	2.77
Q69WH2	Os06g0332800 protein	2.54
Q5W707	Os05g0244600 protein	2.36
Q9FP25	Os01g0303000 protein	2.27
Q10N30	Os03g0284400 protein	2.19
A0A0P0XRR7	Os09g0568900 protein (Fragment)	2.03
Q942Z3	Os01g0934100 protein	1.93
Q6ZL61	Os07g0182100 protein	1.89
Q0DPW6	Os03g0656100 protein (Fragment)	1.75
Q2R176	Os11g0615200 protein	1.70
Q0D6L9	Os07g0467200 protein	1.70
C7J6Y0	Os09g0482780 protein	1.64
Q6K5Y3	Os02g0614200 protein	1.63
A0A0P0Y6D6	Os12g0124000 protein (Fragment)	1.63
Q7XTL6	OSJNBa0070M12.12 protein	1.60
A0A0P0XXQ7	Os10g0568900 protein (Fragment)	1.60
Q69TW4	Os06g0211300 protein	1.57
Q9FTN6	Os01g0106300 protein	1.57
Q6H713	Os02g0170100 protein	1.56
Q6ZBX9	Os08g0562600 protein	1.56
Q6AUG4	Os05g0563550 protein	1.53
A0A0P0V486	Os01g0571100 protein (Fragment)	1.51
A0A0P0WMW0	Os05g0432700 protein (Fragment)	1.50
A0A0P0VEA4	Os02g0131100 protein (Fragment)	0.67
Q5TKJ2	Os05g0429400 protein	0.66
Q0J3S0	Os08g0557100 protein (Fragment)	0.66
Q94DM7	Os01g0962600 protein	0.66
Q7X7H3	OSJNBa0076N16.12 protein	0.65
Q7EYR6	Os07g0262200 protein	0.65
Q8W0I1	Os01g0673600 protein	0.65
Q2QSR7	Os12g0420200 protein	0.65
Q65XN4	Os05g0542900 protein	0.64
Q0JBE3	Os04g0538100 protein (Fragment)	0.63
Q6Z1P2	Os08g0566600 protein	0.62
Q6KA00	Os02g0822600 protein	0.62
Q5Z6P4	Os06g0264800 protein	0.56
Q10NP6	Os03g0263500 protein	0.52
Q8S7H8	Os03g0778100 protein	0.51
Q8LRH2	Os01g0510600 protein	0.49
Q2QWN3	Os12g0189400 protein	0.46
Q75LD8	Os03g0843400 protein	0.46
Q0JCX3	Os04g0445200 protein	0.42
Q2RAK8	Os11g0147800 protein	0.39
Q6AVR1	Expressed protein	0.23
Q0E032	Os02g0581100 protein	0.14
A0A0P0XC80	Os08g0169300 protein	0.04

a: the protein ID come from UniProt databse.

b: T3 represents hybrid wild rice DC907; C3 represents cultivar rice 93–11.

**Fig 4:**
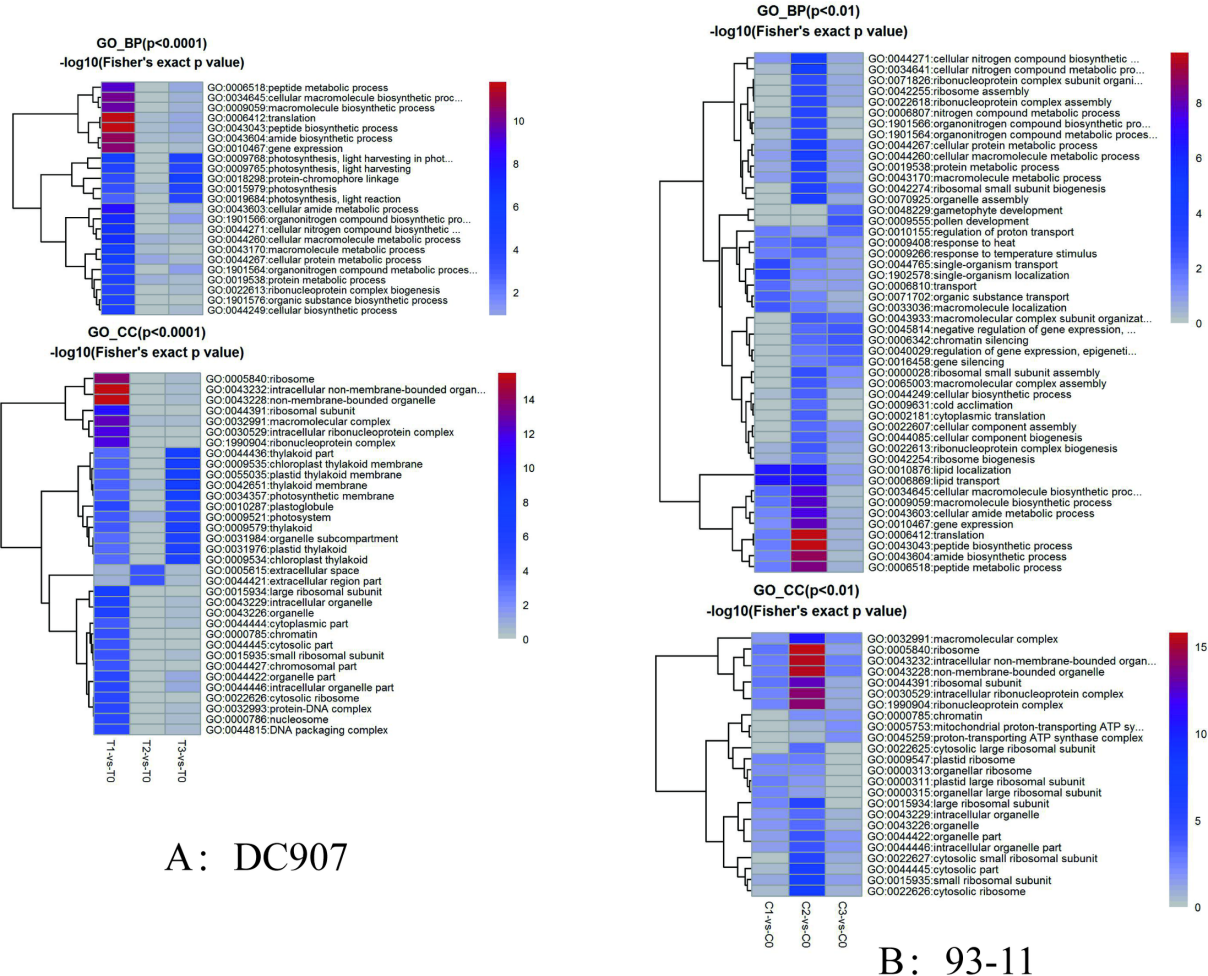
Differentially expressed gene enrichment of 93–11 and DC907 during cold response time course. GO_BP, Gene Ontology Biological Processes. GO_CC: Gene Ontology Cellular Component. 93–11, C0 represents the control group without cold treatment; C1 represents cold treatment for 24 h; C2 represents cold treatment for 72 h; C3 represents cold treatment for 120 h. DC907, T0 represents the control group without cold treatment; T1 represents cold treatment for 24 h; T2 represents cold treatment for 72 h; T3 represents cold treatment for 120 h.

In terms of time frames, the differentially expressed proteins in DC907 contained several functional groups: ATP synthesis, photosystem, reactive oxygen species (ROS), stress response, transcription factors, structural proteins, and cell growth and integrity (S2 Table). Under cold stress, the proteome pattern of hybrid wild rice showed a more sensitive and faster change than cultivar rice, e.g., the vigorous change in protein functional classification was seen at 24 h (Fig 4A), but a similar change occurred in cultivar rice at 72 h (Fig 4B). The delay of cold response in cultivar rice could be a crucial reason for low survival rates. The protein networks of differentially expressed proteins in DC907 show that the centrality of protein change is mainly related to cell structure, stress response, ATP synthesis and photosynthesis (S1 Fig). These networks of functional proteins were generally consistent with those from cultivated rice previously reported using the 2-DE method [13, 14], suggesting that the timely response to cold stress and self-regulation of wild rice is more important than a change in single proteins during cold stress.

### Functional grouping of cold-responding proteins

**Fig 5:**
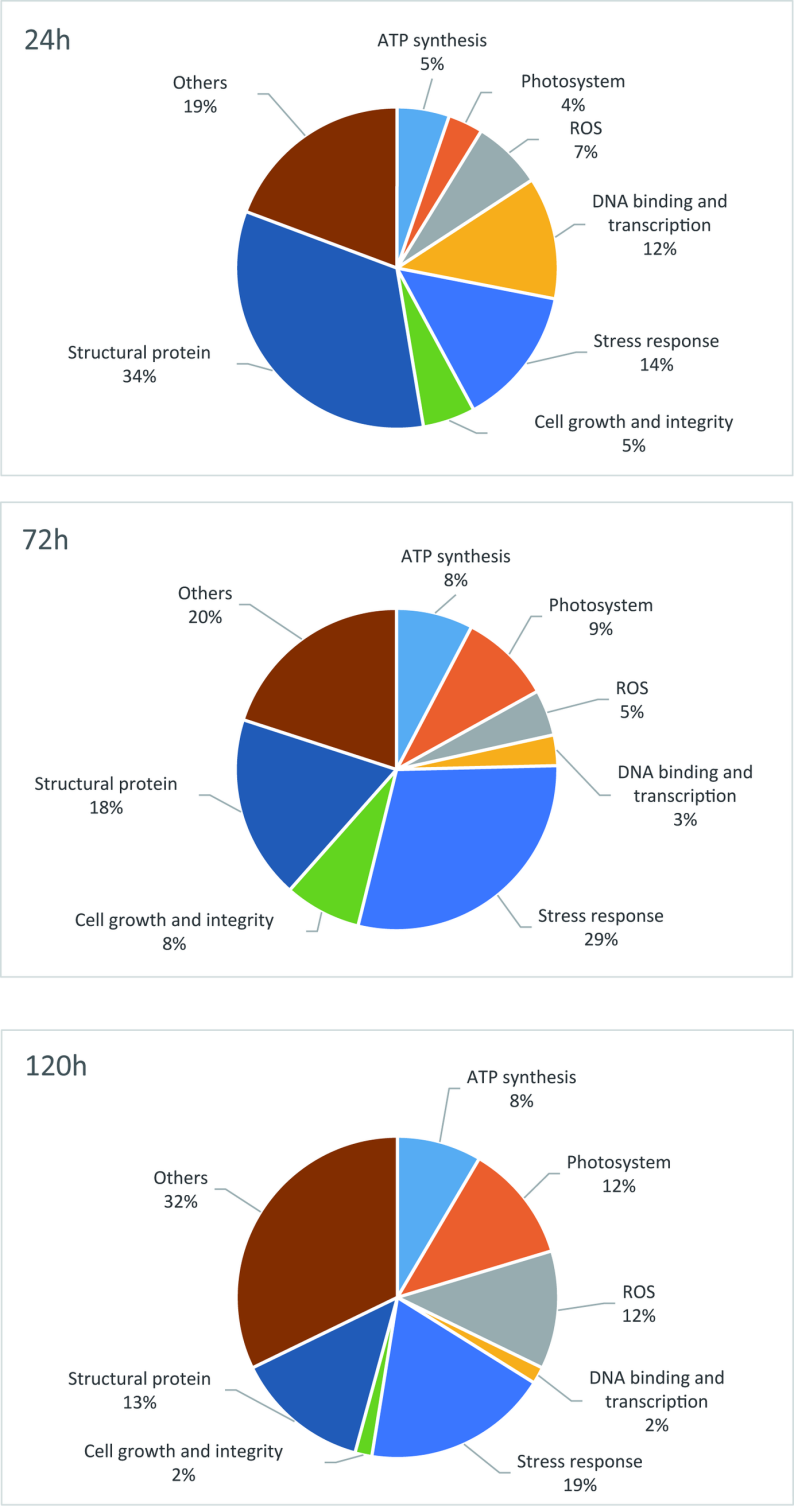
Pie charts of the classifications of functional proteins at different time points.

Differentially expressed proteins between DC907 and 93–11 with assigned functions at all three cold treatment times are summarized in Fig 5. Some of these proteins have been shown or implicated to have functions against cold stress.

#### Non-specific lipid-transfer proteins

Non-specific lipid-transfer proteins (nsLTPs) are a group of small lipophilic proteins that accumulate between the plant epidermis and cell wall [21]. Previous reports have revealed that nsLTPs participate in the process of plant biotic and abiotic stress resistance [22–24]. As seen from the comparative proteomic results between 93–11 and DC907, approximately 10% of the cold-responsive proteins were related to stress response. Although the nsLTPs were both up-regulated in DC907 and 93–11 after cold stress, the comparative expression level of nsLTPs was higher in hybrid wild rice DC907 in the first 24 h and 120 h (Tables 1 and 2), implying that nsLTPs may play an important part in cold tolerance in DC907.

#### Heat shock proteins

Heat shock proteins (HSPs) have been shown to facilitate plant adaptation to environmental changes [25]. A higher level of HSPs, acting as molecular chaperones, may help plants adapt to abnormal temperature, light, drought and salt [26]. As shown in S2 Table, many small HSPs were found to be up-regulated by cold stress in DC907, similar to those found for the cold-tolerance response in *japonica* rice [13].

#### LEA proteins

Up-regulation of late embryogenesis abundant (LEA) proteins was also identified in DC907 (S2 Table). These proteins have been implicated as enhancing plant cold stress tolerance [27–29].

### ROS-related proteins

Reactive oxygen species (ROS) function to oxidize the harmful substrates that may produce and accumulate in the cell. It is now known that ROS-derived signals regulate plant growth, development and stress adaption [30, 31] and are crucial for removing harmful substances from cells and avoiding plant frostbite. Under stress conditions, the ROS level could be increased by 3–10 folds to help the plant adapt to the harsh environment [32]. The ROS related proteins were mainly down-regulated under cold stress in 93–11. The death of 93–11 indicated that harmful substrates may not have been efficiently scavenged in a timely manner and damage of the cells may occur eventually, whereas the expression level of this kind of proteins was relatively stable in DC907 (S1 Table). Thus, insufficiency in ROS may result in plant cell dysfunction and cell death [33, 34].

### Cell structure proteins

A total of 60 differentially expressed proteins related to cell growth, integrity and structure were found (S2 Table). A large amount of ribosomal proteins was found to increase in expression during cold stress both in hybrid DC907 and cultivar rice 93–11, but the time points were different; for 93–11, the highest peak was at 72 h and returned to a normal level at 120 h, and for hybrid DC907, the highest peak was at 24 h and returned to a normal level at 72 h, thus a much faster response in the cold-tolerant hybrid DC907. In *Escherichia coli*, a 70 kDa ribosomal-associated protein (CsdA) was induced when the temperature was shifted from 37 to 15°C, and this protein was further demonstrated to be involved in derepression of HSPs and cell growth at low temperatures [35]. In soybean, three low-temperature inducible ribosomal proteins were found to be increasingly expressed after cold treatment [36].

Dirigent (DIR) proteins were identified as being induced at cold conditions in cultivated rice for the first time in this study. This kind of protein was reported to be involved in lignification and to respond to pathogen infection and abiotic stress in plants [37, 38]. In response to a temperature shift, the expression level of DIRs showed a relatively stable pattern in DC907, similar to hardy plants [39]. In contrast, three DIR proteins were all down-regulated and decreased substantially following the prolonged cold treatment in cultivar rice 93–11. It was reported that a decrease in DIR proteins weakened the process of lignification, which is crucial for the structural integrity of the plant cell wall and cell wall apposition (CWA)-mediated defense [40]. Thus, it is speculated that the decreased expression of DIR proteins in 93–11 results in the vulnerability of *indica* rice to the cold stress since lignification may prevent cells from collapsing and responding to abiotic stress [41].

### Proteins related to photosynthesis and energy metabolism

In the current study, most of the differentially expressed proteins involved in photosynthesis are from photosystem II (PSII), indicating that PSII is more sensitive than photosystem I (PSI) to cold stress [42]. When the cold treatment started, the expression level of chlorophyll a-b binding proteins from the light-harvesting complex (LHC) as a light receptor decreased very fast and was significantly lower in DC907 than in 93–11 (Tables 1–3). This phenomenon is comparable to the previous observation that photoinhibition was a protection mechanism for cold tolerant plants but more significant and protective for cultivar rice in low temperature after long time exposure [43, 44].

Stress-induced inhibition of plant photosynthesis is always coupled with a loss of ATP and ATP synthases [45, 46]. Thus, most of the proteins related to ATP synthesis were found to decrease gradually in DC907 (S2 Table). The decreased ATP synthase resulted in a shortage of ATP, implying a weakened biological activity. The reduction in ATP and ATP synthases directly relates to the reduction in photosynthetic energy captured at low temperatures, and a shortage of energy supply would result in the restriction of normal metabolic processes of plant cells. It is believed that this is a protective mechanism for plant cells under abnormal cold stress [47, 48].

### Interaction networks among cold induced proteins

As show in S1 Fig, protein interaction networks constructed with differentially expressed proteins from hybrid wild rice contain biological processes, molecular functions, and cellular components. In biological processes, proteins that function in photosynthesis, metabolite and energy generation, protein translation, and stress response are the highest positively regulated in DC907; in terms of molecular function, proteins that function in structural activities are most positively regulated in DC907. However, the largest group of regulated proteins at the highest level was in the cellular component domain, including membranes, macromolecule complexes, vacuoles, ribosomes, mitochondria and other organelles. An important observation is that hybrid wild rice responded to cold stress in a more timely manner by mobilizing its signal transduction and self-regulation mechanisms, similar to other cold tolerant plants studied [49].

## Conclusions

**Fig 6:**
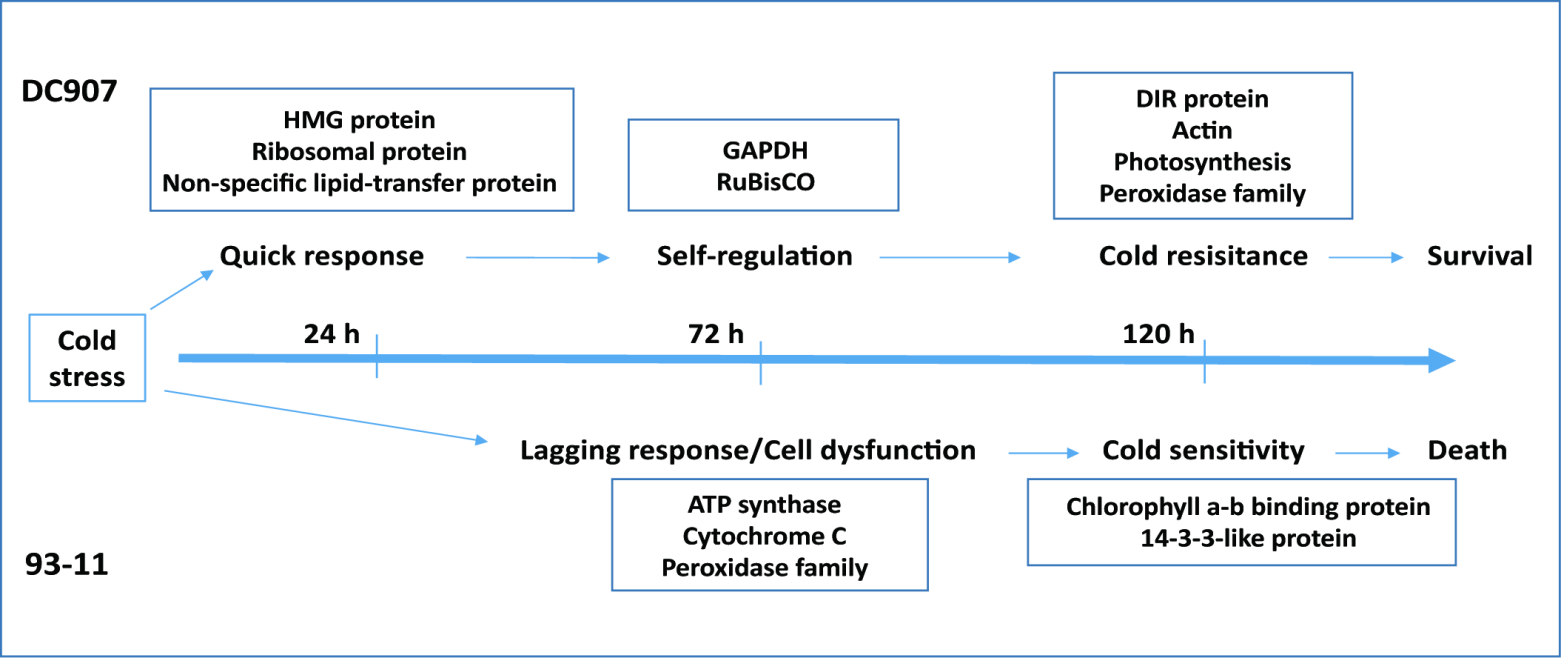
Schematic illustration of cold stress response for DC907 and 93–11.

In this work, the proteomes of cold-resistant DC907 with 93–11 genetic background and the cold-sensitive 93–11 were compared. The protein expression level of several important functional categories, including photosynthesis, energy generation, ROS, cell growth and development, were found to be changed under cold stress. While both DC907 and 93–11 underwent similar alterations in proteomic profiles to cold stress, DC907 responded in a prompter manner in expressing cold-response proteins, maintained a higher level of photosynthesis to power the cells, and possessed a stable and higher level of DIR proteins to prevent the plant from obtaining irreversible cell structure damage induced by ROS activity (Fig 6). Since DC907 carries chromosome fragments of wild rice, future studies should focus on the genetic elements of wild rice that confer the cold tolerant trait in DC907.

## Supporting information

S1 Fig The interaction protein networks of hybrid wild rice DC907. A, biological process; B, molecular function; C, cellular component.
(TIF)

S1 Table Identified information of rice proteins by TMT labeling. (XLSX)

S2 Table List of differentially expressed proteins of hybrid wild rice DC907 during the cold treatment. (DOC)
