# Supplementary figures and images for: Proteomic response of hybrid wild rice to cold stress at the seedling stage
Source: PLoS One. 2018 Jun 7;13(6):e0198675. doi: 10.1371/journal.pone.0198675 (PMC5991693; doi:10.1371/journal.pone.0198675)

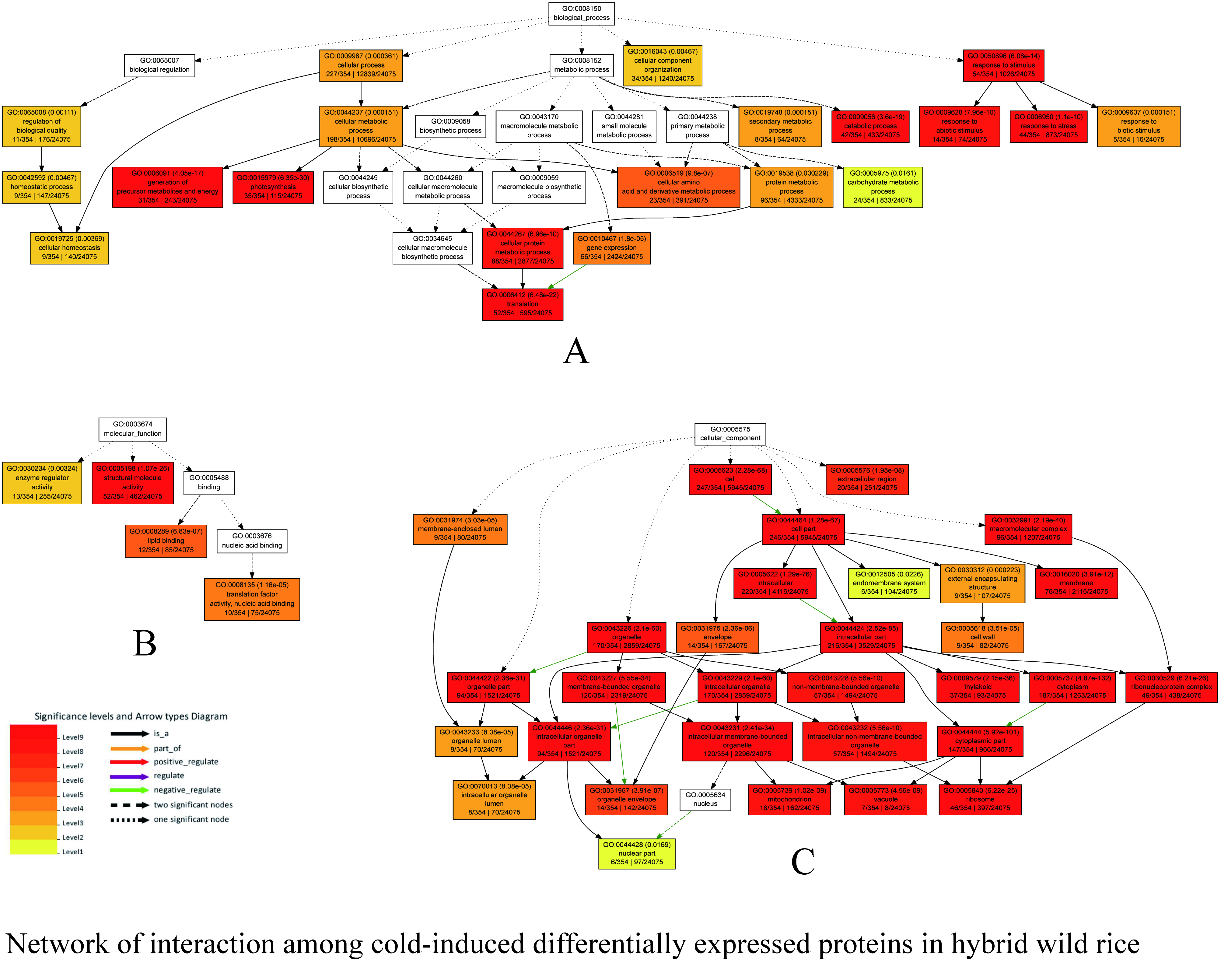

Supplement: S1 Fig — A, biological process; B, molecular function; C, cellular component. (TIF) [file pone.0198675.s001.tif]
