# Supplementary material for: Proteomic response of hybrid wild rice to cold stress at the seedling stage
Source: PLoS One. 2018 Jun 7;13(6):e0198675. doi: 10.1371/journal.pone.0198675 (PMC5991693; doi:10.1371/journal.pone.0198675)
Supplement: S2 Table — (DOC) [file pone.0198675.s003.doc]

**S2 Table. List of differentially expressed proteins of hybrid wild rice DC907 during the cold treatment**

| Protein ID^a^ | Description | T1/T0^b^ | T2/T0 | | T3/T0 | |
| --- | --- | --- | --- | --- | --- | --- |
| **ATP synthesis** |  |  | |  | |  |
| Q7FAM9 | V-type proton ATPase subunit G | 2.01 | 1.76 | | 2.30 | |
| Q6ZG90 | ATP synthase | 1.57 | 1.12 | | 0.95 | |
| Q653V7 | Probable alpha-glucosidase Os06g0675700 | 1.32 | 1.13 | | 1.68 | |
| P48494 | Triosephosphate isomerase, cytosolic | 1.04 | 1.09 | | 1.87 | |
| Q941Z0 | Probable NADPH:quinone oxidoreductase 1 | 0.98 | 1.22 | | 1.57 | |
| P31691 | ADP,ATP carrier protein, mitochondrial | 0.89 | 1.05 | | 0.64 | |
| B9FK36 | Acetyl-CoA carboxylase 2 | 0.89 | 0.80 | | 0.54 | |
| Q53KM3 | Apyrase | 0.89 | 1.23 | | 1.05 | |
| Q84PA4 | ATP synthase B chain, chloroplast, putative, expressed | 0.79 | 0.81 | | 0.77 | |
| Q7X8A1 | Glyceraldehyde-3-phosphate dehydrogenase | 0.76 | 0.87 | | 0.56 | |
| Q9SNK3 | Glyceraldehyde-3-phosphate dehydrogenase | 0.73 | 0.75 | | 0.65 | |
| P0C2Z6 | ATP synthase subunit alpha, chloroplastic | 0.68 | 0.78 | | 0.47 | |
| A0A0P0WP33 | Phosphoglycerate kinase | 0.65 | 0.90 | | 0.55 | |
| Q6K5G8 | Glyceraldehyde-3-phosphate dehydrogenase 3, cytosolic | 0.65 | 1.01 | | 0.69 | |
| Q0JGZ6 | Fructokinase-1 | 0.64 | 0.93 | | 0.60 | |
| Q0JHF8 | Fructose-1,6-bisphosphatase, cytosolic | 0.64 | 1.00 | | 0.54 | |
| P12085 | ATP synthase subunit beta, chloroplastic | 0.60 | 0.80 | | 0.65 | |
| P0C2Z0 | ATP synthase subunit b, chloroplastic | 0.58 | 0.83 | | 0.51 | |
| Q40677 | Fructose-bisphosphate aldolase, chloroplastic | 0.49 | 0.92 | | 0.53 | |
| **Photosystem** |  |  | |  | |  |
| Q6ESZ6 | Cytochrome b-c1 complex subunit 6 | 1.77 | 1.34 | | 1.24 | |
| Q0DI31 | Cytochrome c | 1.39 | 1.07 | | 1.82 | |
| Q9SXV0 | Cytochrome c oxidase subunit 6b | 1.32 | 1.30 | | 1.79 | |
| P49100 | Cytochrome b5 | 1.10 | 0.98 | | 0.64 | |
| Q69S39 | Cytochrome b6-f complex iron-sulfur subunit, chloroplastic | 1.02 | 1.40 | | 0.78 | |
| P0C389 | Cytochrome f | 0.86 | 0.85 | | 0.57 | |
| Q0DG05 | Photosystem I reaction center subunit VI, chloroplastic | 0.85 | 1.28 | | 1.28 | |
| P93431 | Ribulose bisphosphate carboxylase/oxygenase activase, chloroplastic | 0.82 | 0.92 | | 0.67 | |
| P0C319 | Cytochrome b6-f complex subunit 4 | 0.80 | 0.98 | | 0.55 | |
| Q0JG75 | Photosystem II reaction center PSB28 protein, chloroplastic | 0.80 | 0.84 | | 1.29 | |
| P0C355 | Photosystem I P700 chlorophyll a apoprotein A1 | 0.77 | 0.85 | | 0.59 | |
| P0C364 | Photosystem II CP47 reaction center protein | 0.74 | 0.82 | | 0.60 | |
| Q6ZFJ3 | Ferredoxin--NADP reductase | 0.71 | 0.82 | | 0.56 | |
| Q0J8M2 | Ferredoxin-1, chloroplastic | 0.71 | 1.08 | | 1.09 | |
| Q7XC09 | Chloroplast chaperonin 10, putative, expressed | 0.67 | 0.94 | | 1.03 | |
| P18566 | Ribulose bisphosphate carboxylase small chain A, chloroplastic | 0.66 | 1.11 | | 0.40 | |
| P0C512 | Ribulose bisphosphate carboxylase large chain | 0.66 | 0.93 | | 0.52 | |
| Q84PB4 | Chloroplast photosystem I reaction center subunit II-like protein | 0.65 | 0.86 | | 0.79 | |
| Q69RJ0 | Ferredoxin-dependent glutamate synthase, chloroplastic | 0.64 | 0.83 | | 0.52 | |
| Q6ZF30 | Chlorophyll a-b binding protein, chloroplastic | 0.64 | 0.98 | | 0.52 | |
| P0C370 | Cytochrome b559 subunit alpha | 0.63 | 0.71 | | 0.47 | |
| P0C401 | Cytochrome b559 subunit beta | 0.62 | 0.84 | | 0.56 | |
| Q53N83 | Chlorophyll a-b binding protein, chloroplastic | 0.60 | 0.78 | | 0.46 | |
| Q6K471 | Ferredoxin-thioredoxin reductase catalytic chain, chloroplastic | 0.58 | 0.92 | | 0.96 | |
| P0C422 | Photosystem II reaction center protein H | 0.57 | 0.66 | | 0.49 | |
| Q10HD0 | Chlorophyll a-b binding protein, chloroplastic | 0.57 | 0.87 | | 0.49 | |
| Q6H748 | Chlorophyll a-b binding protein, chloroplastic | 0.53 | 0.87 | | 0.53 | |
| Q6Z411 | Chlorophyll a-b binding protein, chloroplastic | 0.53 | 0.83 | | 0.40 | |
| Q7XV11 | Chlorophyll a-b binding protein, chloroplastic | 0.47 | 0.79 | | 0.33 | |
| Q5ZA98 | Chlorophyll a-b binding protein, chloroplastic | 0.41 | 0.87 | | 0.25 | |
| **ROS** |  |  | |  | |  |
| Q7XSU2 | Peroxidase | 1.94 | 1.42 | | 1.43 | |
| P28757 | Superoxide dismutase [Cu-Zn] 2 | 1.87 | 1.62 | | 2.45 | |
| Q9AS12 | Peroxidase | 1.57 | 1.29 | | 2.01 | |
| P37834 | Peroxidase 1 | 1.50 | 0.90 | | 1.63 | |
| Q7XSV2 | Peroxidase | 1.40 | 1.03 | | 1.33 | |
| A0A0P0V2C2 | Peroxidase (Fragment) | 1.23 | 0.73 | | 1.71 | |
| Q6ZFU6 | Thioredoxin reductase NTRB | 1.22 | 1.17 | | 1.62 | |
| Q5JMS4 | Peroxidase | 1.20 | 1.13 | | 1.42 | |
| Q6ZJ08 | Monodehydroascorbate reductase | 1.13 | 1.30 | | 1.61 | |
| P55142 | Glutaredoxin-C6 | 1.07 | 1.09 | | 1.51 | |
| Q75IS1 | Peroxidase | 1.05 | 1.05 | | 1.64 | |
| Q9SDD6 | Peroxiredoxin-2F, mitochondrial | 0.97 | 1.01 | | 0.60 | |
| Q0E4K1 | Catalase isozyme A | 0.96 | 1.10 | | 0.61 | |
| P0C5D1 | 1-Cys peroxiredoxin B | 0.95 | 0.65 | | 1.48 | |
| Q7F1U0 | Peroxidase | 0.94 | 0.98 | | 1.29 | |
| Q0D840 | Thioredoxin H1 | 0.93 | 1.06 | | 2.10 | |
| Q75KD7 | Allene oxide cyclase, chloroplastic | 0.86 | 0.92 | | 0.60 | |
| Q6Z4A7 | Probable 5'-adenylylsulfate reductase 1, chloroplastic | 0.83 | 0.91 | | 0.63 | |
| Q69P84 | Aldehyde dehydrogenase | 0.83 | 0.95 | | 0.67 | |
| Q10S82 | Catalase | 0.82 | 1.01 | | 0.63 | |
| Q7XKD0 | Thioredoxin X, chloroplastic | 0.75 | 0.91 | | 1.53 | |
| Q9ZP20 | Thioredoxin M5, chloroplastic | 0.75 | 0.94 | | 0.83 | |
| Q2QRV3 | Alpha-dioxygenase, putative, expressed | 0.73 | 0.95 | | 0.57 | |
| Q5Z9Z3 | Thioredoxin-like protein Clot | 0.73 | 0.85 | | 0.65 | |
| Q7F8S5 | Peroxiredoxin-2E-2, chloroplastic | 0.71 | 0.99 | | 1.00 | |
| Q6H759 | Copper chaperone homolog CCH | 0.71 | 0.84 | | 1.21 | |
| Q0D5P8 | Oxygen-evolving enhancer protein 3, chloroplastic | 0.68 | 1.00 | | 0.84 | |
| Q6ER94 | 2-Cys peroxiredoxin BAS1, chloroplastic | 0.66 | 0.98 | | 1.05 | |
| Q6YT73 | Peroxisomal (S)-2-hydroxy-acid oxidase GLO5 | 0.63 | 0.81 | | 0.63 | |
| Q8H4J8 | Aldo/keto reductase family-like protein | 0.59 | 0.72 | | 0.58 | |
| Q8S091 | Thioredoxin F, chloroplastic | 0.58 | 0.69 | | 0.70 | |
| **DNA binding and transcription** | |  |  | |  | |
| Q94HA1 | Gibberellin stimulated transcript related protein 1 | 4.17 | 2.09 | | 0.88 | |
| Q5Z7N3 | HMG protein | 3.29 | 1.48 | | 0.89 | |
| Q7XQK2 | HMG protein | 1.87 | 1.19 | | 0.73 | |
| Q6YTY3 | PHD finger protein ALFIN-LIKE 9 | 1.25 | 1.14 | | 0.50 | |
| Q7GD79 | GTP-binding nuclear protein Ran-2 | 1.02 | 0.79 | | 0.61 | |
| Q2R1S1 | Harpin binding protein 1, putative, expressed | 0.95 | 0.88 | | 1.20 | |
| Q5N7L5 | Met-tRNAi formyl transferase-like | 0.86 | 0.90 | | 0.61 | |
| **Stress response** |  |  |  | |  | |
| Q5QM60 | Non-specific lipid-transfer protein | 3.21 | 1.11 | | 1.64 | |
| Q2QYL0 | Non-specific lipid-transfer protein | 2.20 | 1.19 | | 1.80 | |
| Q2QYL3 | Non-specific lipid-transfer protein 3 | 2.10 | 0.60 | | 1.89 | |
| Q7G2B5 | Nonspecific lipid-transfer protein 2, putative, expressed | 2.10 | 1.28 | | 3.00 | |
| A0A0P0WNP9 | Non-specific lipid-transfer protein (Fragment) | 2.07 | 1.00 | | 1.82 | |
| Q7XBA6 | Non-specific lipid-transfer protein | 1.94 | 1.21 | | 1.74 | |
| Q0E4A8 | 18.9 kDa heat shock protein | 1.64 | 0.92 | | 1.53 | |
| Q6K3Y6 | NOI protein | 1.57 | 1.26 | | 1.59 | |
| Q6ESR4 | Dehydration-stress inducible protein 1 | 1.50 | 1.23 | | 1.40 | |
| Q2QYK8 | Non-specific lipid-transfer protein | 1.50 | 1.11 | | 1.77 | |
| Q6ZBZ2 | Germin-like protein 8-14 | 1.47 | 1.24 | | 5.29 | |
| Q7XJ39 | Non-specific lipid-transfer protein 2A | 1.47 | 1.39 | | 2.58 | |
| P25776 | Oryzain alpha chain | 1.44 | 1.37 | | 1.90 | |
| Q8S3P3 | DUF26-like protein | 1.20 | 0.99 | | 2.25 | |
| Q656V1 | Peptidylprolyl isomerase | 1.16 | 0.68 | | 0.94 | |
| Q69U53 | MAP3K-like protein | 1.14 | 1.18 | | 1.73 | |
| P25778 | Oryzain gamma chain | 1.12 | 0.65 | | 1.50 | |
| Q42456 | Aspartic proteinase oryzasin-1 | 1.10 | 1.03 | | 1.36 | |
| P0C5A4 | Late embryogenesis abundant protein, group 3 | 1.10 | 2.45 | | 2.54 | |
| Q0JMY8 | Salt stress-induced protein | 1.06 | 0.98 | | 1.32 | |
| Q5VQK9 | Protein CHLOROPLAST ENHANCING STRESS TOLERANCE, chloroplastic | 1.01 | 0.99 | | 0.61 | |
| Q8H3I7 | 10 kDa chaperonin | 1.01 | 1.07 | | 1.53 | |
| Q10KY5 | 10 kDa chaperonin, putative, expressed | 0.95 | 0.97 | | 1.64 | |
| Q84Q77 | 17.9 kDa class I heat shock protein | 0.91 | 0.85 | | 1.01 | |
| Q07661 | Nucleoside diphosphate kinase 1 | 0.89 | 0.92 | | 1.56 | |
| Q6Z7V2 | 24.1 kDa heat shock protein, mitochondrial | 0.88 | 1.01 | | 1.29 | |
| Q5Z9N8 | 90 kDa heat shock protein | 0.85 | 0.81 | | 0.65 | |
| Q7Y139 | Huntingtin interacting protein K, putative, expressed | 0.85 | 1.05 | | 1.67 | |
| Q0J4P2 | Heat shock protein 81-1 | 0.83 | 0.94 | | 0.50 | |
| Q6ZIT9 | Peptidylprolyl isomerase | 0.80 | 1.05 | | 1.22 | |
| Q2R2W2 | 14-3-3-like protein GF14-D | 0.76 | 0.76 | | 0.53 | |
| Q07078 | Heat shock protein 81-3 | 0.72 | 0.91 | | 0.43 | |
| Q6ZKC0 | 14-3-3-like protein GF14-C | 0.70 | 0.97 | | 0.65 | |
| Q652V8 | 16.0 kDa heat shock protein, peroxisomal | 0.66 | 0.94 | | 0.90 | |
| P55857 | Small ubiquitin-related modifier 1 | 0.63 | 0.91 | | 1.05 | |
| Q2QU06 | 60 kDa chaperonin alpha subunit | 0.61 | 0.83 | | 0.67 | |
| **Cell growth and integrity** | |  |  | |  | |
| Q2QZU5 | Auxin-repressed protein-like protein ARP1, putative, expressed | 1.95 | 1.19 | | 1.13 | |
| Q7Y1Z0 | Chitinase 5 | 1.75 | 1.22 | | 1.27 | |
| Q94LR4 | Expansin-B4 | 1.75 | 1.35 | | 1.16 | |
| O24230 | Expansin-B2 | 1.70 | 1.40 | | 1.37 | |
| P35681 | Translationally-controlled tumor protein homolog | 1.66 | 1.09 | | 1.76 | |
| Q942D4 | BURP domain-containing protein 3 | 1.63 | 1.12 | | 1.58 | |
| Q53NQ9 | Dirigent protein | 1.56 | 1.09 | | 1.25 | |
| Q5VND6 | Nucleosome assembly protein 1;1 | 1.53 | 1.13 | | 1.27 | |
| O04138 | Chitinase 4 | 1.44 | 1.25 | | 1.54 | |
| Q7XZG9 | Peptidyl-prolyl cis-trans isomerase | 1.19 | 1.00 | | 1.57 | |
| Q5QLS1 | Arabinogalactan protein-like | 1.22 | 1.01 | | 2.15 | |
| Q8GTK0 | Starch synthase, chloroplastic/amyloplastic | 1.13 | 1.14 | | 0.88 | |
| Q8H8C7 | Chitin elicitor-binding protein | 0.99 | 0.96 | | 1.66 | |
| Q84TB3 | Actin-depolymerizing factor 4 | 0.93 | 0.89 | | 1.52 | |
| Q6YVJ0 | 9-cis-epoxycarotenoid dioxygenase 1 | 0.82 | 0.66 | | 0.66 | |
| A0A0P0Y7F4 | Dirigent protein (Fragment) | 0.69 | 0.94 | | 0.80 | |
| Q0E1D7 | Flowering-promoting factor 1-like protein 3 | 0.68 | 0.67 | | 0.89 | |
| A3BLC3 | Ribosome-recycling factor, chloroplastic | 0.62 | 0.97 | | 0.87 | |
| Q5Z974 | ATP-dependent zinc metalloprotease FTSH 1, chloroplastic | 0.61 | 0.76 | | 0.53 | |
| Q67VQ4 | BolA-like | 0.59 | 0.91 | | 0.78 | |
| Q0JEF5 | Flowering-promoting factor 1-like protein 4 | 0.46 | 0.77 | | 0.77 | |
| **Structural protein** | |  |  | |  | |
| P31674 | 40S ribosomal protein S15 | 5.28 | 1.57 | | 0.84 | |
| Q6YY64 | 60S ribosomal protein L6 | 3.67 | 1.25 | | 0.77 | |
| Q53QG2 | 40S ribosomal protein S25, putative, expressed | 3.50 | 1.46 | | 1.11 | |
| A3AGM4 | Histone H2B.1 | 3.36 | 1.43 | | 1.24 | |
| Q7XR19 | 60S ribosomal protein L6 | 3.31 | 1.19 | | 0.74 | |
| Q2QNF3 | 60S ribosomal protein L2 | 3.07 | 1.11 | | 0.88 | |
| Q0IQF7 | 40S ribosomal protein S16 | 2.75 | 1.34 | | 0.67 | |
| Q7XEQ3 | 40S ribosomal protein S17-4, putative, expressed | 2.70 | 1.12 | | 0.87 | |
| Q84M35 | 40S ribosomal protein S2, putative, expressed | 2.58 | 1.39 | | 0.68 | |
| Q9ZST1 | 30S ribosomal protein S17, chloroplastic | 2.34 | 1.22 | | 0.97 | |
| P49398 | 40S ribosomal protein S4 | 2.21 | 1.18 | | 0.74 | |
| P12153 | 30S ribosomal protein S19, chloroplastic | 2.11 | 0.93 | | 0.93 | |
| Q10PV6 | 50S ribosomal protein L15, chloroplast, putative, expressed | 2.08 | 1.38 | | 0.84 | |
| Q8SAY0 | 50S ribosomal protein L18, chloroplastic | 2.06 | 1.43 | | 0.45 | |
| Q6ZL42 | Probable histone H2A.2 | 2.04 | 1.32 | | 0.80 | |
| Q10L93 | 50S ribosomal protein L6, putative, expressed | 1.97 | 1.33 | | 0.83 | |
| Q6AUF5 | 60S ribosomal protein L18a | 1.90 | 1.08 | | 1.03 | |
| Q9AV77 | 60S ribosomal protein L17 | 1.73 | 1.00 | | 0.50 | |
| Q84NJ4 | Probable histone H2A.3 | 1.67 | 1.16 | | 0.67 | |
| Q7XUC9 | Histone H4 | 1.66 | 1.08 | | 0.51 | |
| Q10MS5 | 40S ribosomal protein S7, putative, expressed | 1.61 | 1.19 | | 1.02 | |
| P40978 | 40S ribosomal protein S19 | 1.51 | 1.08 | | 1.01 | |
| Q851P9 | Histone-like protein | 1.50 | 1.37 | | 1.00 | |
| P49210 | 60S ribosomal protein L9 | 1.37 | 1.17 | | 0.67 | |
| Q9SDG6 | 60S ribosomal protein L30 | 1.35 | 1.39 | | 0.76 | |
| Q6K5R5 | 40S ribosomal protein S27 | 1.18 | 1.15 | | 0.61 | |
| Q2R4A1 | 40S ribosomal protein S5, putative, expressed | 1.03 | 0.91 | | 0.63 | |
| Q2R1J8 | 40S ribosomal protein S9, putative, expressed | 1.01 | 1.02 | | 0.65 | |
| Q76FS3 | Tubulin beta-6 chain | 1.00 | 0.95 | | 0.66 | |
| Q75G91 | 40S ribosomal protein S3, putative, expressed | 1.00 | 1.00 | | 0.54 | |
| Q10NM5 | 50S ribosomal protein L4, chloroplast, putative, expressed | 0.91 | 0.95 | | 0.48 | |
| P37832 | Tubulin beta-7 chain | 0.87 | 0.75 | | 0.63 | |
| Q2QYW2 | Clathrin heavy chain 2 | 0.87 | 0.80 | | 0.64 | |
| A0A0P0WK98 | Ribosomal protein L15 (Fragment) | 0.82 | 0.84 | | 0.53 | |
| P35684 | 60S ribosomal protein L3 | 0.81 | 0.91 | | 0.61 | |
| Q10NB4 | Clathrin assembly protein, putative, expressed | 0.76 | 0.83 | | 0.65 | |
| Q10DV7 | Actin-1 | 0.66 | 1.33 | | 0.53 | |
| O22386 | 50S ribosomal protein L12, chloroplastic | 0.60 | 1.33 | | 0.82 | |
| P0C440 | 50S ribosomal protein L14, chloroplastic | 0.57 | 0.94 | | 0.56 | |
| **Others** |  |  | |  | |  |
| Q9LGE6 | Probable U6 snRNA-associated Sm-like protein LSm4 | 1.88 | 1.24 | | 1.25 | |
| Q10N98 | 33 kDa secretory protein, putative, expressed | 1.76 | 2.93 | | 2.50 | |
| Q9AUW3 | Eukaryotic translation initiation factor 5A | 1.70 | 1.19 | | 1.00 | |
| Q10LV9 | Eukaryotic translation initiation factor 2 beta subunit, putative, expressed | 1.57 | 1.09 | | 0.83 | |
| P42862 | Glucose-6-phosphate isomerase, cytosolic A | 1.61 | 1.13 | | 0.99 | |
| Q6AUR2 | Nitrogen regulatory protein P-II homolog | 1.55 | 1.36 | | 1.53 | |
| Q688X1 | Eukaryotic translation initiation factor 3 subunit D | 1.55 | 0.85 | | 0.74 | |
| Q6F4N5 | Aspartyl protease 25 | 1.44 | 0.92 | | 1.20 | |
| Q69T99 | Glucose-1-phosphate adenylyltransferase | 1.37 | 0.79 | | 0.85 | |
| Q6ZCF0 | Probable gamma-aminobutyrate transaminase 3, mitochondrial | 1.35 | 1.30 | | 1.02 | |
| Q6K7H2 | UMP-CMP kinase 4 | 1.28 | 1.12 | | 1.54 | |
| Q10J94 | Cysteine proteinase inhibitor 8 | 1.27 | 1.44 | | 1.68 | |
| Q7XCS3 | Cys/Met metabolism PLP-dependent enzyme family protein, expressed | 1.24 | 1.43 | | 2.63 | |
| Q8LMR0 | Phosphoserine aminotransferase | 1.19 | 1.16 | | 2.00 | |
| P42211 | Aspartic proteinase | 1.12 | 0.96 | | 0.86 | |
| Q6YPF1 | Fructose-bisphosphate aldolase | 1.11 | 1.30 | | 2.11 | |
| Q6Z0G0 | GrpE protein homolog | 1.10 | 1.03 | | 1.51 | |
| O64437 | Inositol-3-phosphate synthase | 1.07 | 1.49 | | 2.08 | |
| Q8LJH1 | Hexosyltransferase | 1.02 | 1.03 | | 1.30 | |
| Q0DJ99 | Coatomer subunit delta-2 | 1.00 | 0.99 | | 0.54 | |
| Q5Z627 | Elongation factor 1-gamma 3 | 0.99 | 0.96 | | 0.46 | |
| A0A0P0XBI6 | Tryptophan synthase (Fragment) | 0.97 | 1.34 | | 2.04 | |
| P20907 | Cysteine proteinase inhibitor 2 | 0.94 | 1.15 | | 1.64 | |
| A0A0P0V9F2 | Cysteine proteinase inhibitor (Fragment) | 0.93 | 0.77 | | 1.76 | |
| Q5ZAF2 | Glycosyltransferase | 0.91 | 1.90 | | 2.81 | |
| Q0IPE5 | 2-dehydro-3-deoxyphosphooctonate aldolase, putative, expressed (Fragment) | 0.89 | 1.10 | | 0.58 | |
| Q10PB3 | Translocase of chloroplast | 0.84 | 0.87 | | 0.58 | |
| Q6YW46 | Elongation factor 1-gamma 2 | 0.84 | 0.83 | | 0.53 | |
| Q6H4L2 | Elongation factor 2 | 0.84 | 0.79 | | 0.62 | |
| Q8S0J7 | Probable membrane-associated 30 kDa protein, chloroplastic | 0.84 | 0.92 | | 0.87 | |
| A0A0P0Y1Y5 | Adenosylhomocysteinase (Fragment) | 0.83 | 0.90 | | 0.58 | |
| Q75HE6 | Probable methylenetetrahydrofolate reductase | 0.83 | 0.91 | | 0.64 | |
| Q7XV22 | Gamma-glutamylcyclotransferase | 0.81 | 1.01 | | 1.07 | |
| Q2QLY4 | 5-methyltetrahydropteroyltriglutamate--homocysteine methyltransferase 2 | 0.78 | 0.81 | | 0.58 | |
| P14655 | Glutamine synthetase, chloroplastic | 0.78 | 0.92 | | 0.93 | |
| Q10I42 | HAD-superfamily hydrolase, subfamily IA, variant 3 containing protein, expressed | 0.77 | 1.02 | | 0.63 | |
| Q7XBY4 | Mitochondrial carnitine/acylcarnitine carrier, putative, expressed | 0.77 | 0.88 | | 0.91 | |
| B7EA73 | Puromycin-sensitive aminopeptidase | 0.76 | 0.90 | | 0.65 | |
| Q943W5 | Tyrosine--tRNA ligase | 0.75 | 1.10 | | 0.55 | |
| Q0DWH1 | Alcohol dehydrogenase class-3 | 0.74 | 0.98 | | 0.63 | |
| P15280 | Glucose-1-phosphate adenylyltransferase small subunit, chloroplastic/amyloplastic | 0.74 | 0.89 | | 0.53 | |
| Q6K6Q1 | Phenylalanine ammonia-lyase | 0.74 | 0.87 | | 0.67 | |
| Q10D68 | Serine hydroxymethyltransferase | 0.73 | 0.91 | | 0.85 | |
| Q6ZJX8 | 33-kDa secretory protein | 0.72 | 0.70 | | 1.40 | |
| Q7XXS4 | Thiamine thiazole synthase, chloroplastic | 0.71 | 0.85 | | 0.55 | |
| A0A0P0W1T3 | Lipoxygenase (Fragment) | 0.70 | 0.98 | | 0.87 | |
| P14717 | Phenylalanine ammonia-lyase | 0.69 | 0.87 | | 0.63 | |
| Q69Q02 | 4-alpha-glucanotransferase DPE2 | 0.69 | 0.78 | | 0.55 | |
| Q7F2G3 | Carbonic anhydrase | 0.68 | 1.20 | | 0.52 | |
| Q0DYB1 | Soluble inorganic pyrophosphatase | 0.68 | 0.72 | | 1.52 | |
| Q6ESI7 | Tripeptidyl-peptidase 2 | 0.68 | 0.84 | | 0.58 | |
| Q8S718 | Glutathione S-transferase GSTU6, putative, expressed | 0.67 | 0.76 | | 0.84 | |
| B7FAE9 | Glutathione peroxidase | 0.66 | 0.97 | | 0.85 | |
| Q2QP54 | Elongation factor Ts, mitochondrial | 0.63 | 0.89 | | 0.65 | |
| A3C6G9 | Glycine cleavage system H protein, mitochondrial | 0.63 | 1.05 | | 0.89 | |
| Q53PA7 | 2-oxoglutarate/malate translocator (Clone OMT103), mitochondrial membrane-proso millet | 0.62 | 0.98 | | 0.66 | |
| Q10MQ2 | Probable LL-diaminopimelate aminotransferase, chloroplastic | 0.60 | 0.87 | | 0.60 | |
| Q9XEA6 | Cysteine synthase | 0.56 | 0.84 | | 0.88 | |
| **Unknown** |  |  | |  | |  |
| Q0DWC5 | Os02g0821200 protein (Fragment) | 4.83 | 1.10 | | 0.77 | |
| Q6K1W6 | Os09g0258600 protein | 4.24 | 1.21 | | 0.95 | |
| Q6ZIA1 | Os08g0530200 protein | 4.00 | 1.45 | | 1.05 | |
| A0A0P0VUA6 | Os03g0200500 protein (Fragment) | 3.79 | 1.74 | | 0.99 | |
| Q6ZLB8 | Os07g0180900 protein | 3.28 | 1.13 | | 0.82 | |
| Q6YZI5 | Os08g0558900 protein | 2.95 | 1.22 | | 1.21 | |
| A0A0P0XW06 | Os10g0465800 protein (Fragment) | 2.83 | 0.97 | | 1.00 | |
| Q2R176 | Os11g0615200 protein | 2.73 | 1.77 | | 2.09 | |
| Q5TKP2 | Os05g0541900 protein | 2.61 | 1.22 | | 0.82 | |
| A0A0N7KGC5 | Os02g0821800 protein | 2.47 | 1.12 | | 0.90 | |
| Q0IVE4 | Os10g0576000 protein (Fragment) | 2.43 | 1.20 | | 1.38 | |
| Q8SA35 | Os01g0659200 protein | 2.25 | 1.00 | | 0.83 | |
| Q2QND9 | Expressed protein | 2.24 | 1.04 | | 1.45 | |
| Q84ZP1 | Os07g0208000 protein | 2.13 | 1.24 | | 0.77 | |
| Q2RBP5 | Os11g0103900 protein | 2.11 | 1.63 | | 0.98 | |
| A0A0P0VUL0 | Os03g0210600 protein (Fragment) | 2.05 | 1.32 | | 1.12 | |
| Q5Z9Z8 | Os06g0319700 protein | 2.04 | 1.03 | | 0.79 | |
| A0A0P0X4N7 | Os07g0243150 protein (Fragment) | 2.02 | 2.21 | | 1.01 | |
| Q9FTY4 | Os01g0104400 protein | 1.96 | 1.33 | | 1.03 | |
| Q0JBZ7 | Os04g0501000 protein | 1.94 | 0.69 | | 0.67 | |
| A0A0P0WRW9 | Os05g0597200 protein (Fragment) | 1.92 | 1.24 | | 1.28 | |
| A0A0P0VYT7 | Os03g0417800 protein (Fragment) | 1.87 | 1.28 | | 1.19 | |
| Q8H3M0 | Os08g0428800 protein | 1.81 | 1.12 | | 0.88 | |
| Q0E446 | Os02g0137200 protein (Fragment) | 1.81 | 1.30 | | 0.97 | |
| A0A0P0WG50 | Os04g0665500 protein | 1.79 | 0.96 | | 1.52 | |
| Q5N8I8 | Os01g0700000 protein | 1.78 | 1.53 | | 1.67 | |
| A0A0P0XK49 | Os09g0326900 protein (Fragment) | 1.78 | 1.05 | | 1.37 | |
| Q9FTR9 | Os01g0127600 protein | 1.71 | 1.23 | | 1.13 | |
| Q6ZLP6 | Os07g0150500 protein | 1.75 | 1.26 | | 1.75 | |
| Q6H7T1 | Os02g0162500 protein | 1.69 | 1.07 | | 0.86 | |
| Q6KA00 | Os02g0822600 protein | 1.65 | 1.36 | | 0.67 | |
| Q5VRC9 | Os01g0179300 protein | 1.65 | 1.13 | | 1.05 | |
| Q0J0H6 | Os09g0509000 protein | 1.56 | 1.02 | | 1.51 | |
| Q5N865 | Os01g0897200 protein | 1.54 | 1.22 | | 0.98 | |
| Q7XTY1 | OSJNBa0019K04.16 protein | 1.53 | 0.95 | | 1.00 | |
| Q5Z4M1 | Os06g0308300 protein | 1.53 | 0.91 | | 0.65 | |
| B9GC17 | Os12g0165900 protein | 1.51 | 1.00 | | 0.91 | |
| Q75LJ7 | Os03g0836200 protein | 1.51 | 1.16 | | 1.97 | |
| Q7XRA0 | OSJNBb0085F13.15 protein | 1.51 | 1.57 | | 1.31 | |
| Q6K7A3 | Os02g0469600 protein | 1.51 | 1.52 | | 2.45 | |
| Q65XN5 | Os05g0542800 protein | 1.50 | 1.18 | | 1.45 | |
| Q0JN91 | Os01g0314800 protein | 1.46 | 2.21 | | 4.07 | |
| Q0DK70 | Os05g0188100 protein | 1.42 | 1.22 | | 1.99 | |
| Q5ZDH9 | Os01g0139200 protein | 1.41 | 1.27 | | 1.68 | |
| Q6K5Y3 | Os02g0614200 protein | 1.41 | 1.48 | | 1.83 | |
| Q5VPQ6 | Os06g0119600 protein | 1.38 | 1.23 | | 1.16 | |
| Q7XDI5 | Os10g0470900 protein | 1.38 | 1.10 | | 1.56 | |
| Q5QNJ0 | Os01g0214600 protein | 1.37 | 1.09 | | 1.32 | |
| Q0DJF8 | Os05g0292200 protein | 1.35 | 1.45 | | 1.73 | |
| A0A0P0W5Q6 | Os03g0854400 protein | 1.33 | 1.08 | | 1.59 | |
| Q941F5 | Os11g0592200 protein | 1.30 | 1.28 | | 1.68 | |
| A0A0P0Y573 | Os11g0673200 protein | 1.28 | 1.16 | | 1.10 | |
| Q7XCL2 | Os10g0542200 protein | 1.28 | 1.15 | | 1.73 | |
| Q8LHN4 | Os07g0631900 protein | 1.27 | 1.62 | | 1.69 | |
| Q5W6H1 | Os05g0350500 protein | 1.27 | 1.01 | | 0.66 | |
| Q5W707 | Os05g0244600 protein | 1.26 | 1.12 | | 2.47 | |
| Q6ZGV9 | Os02g0717300 protein | 1.25 | 1.03 | | 1.55 | |
| Q2QNS7 | Os12g0555500 protein | 1.24 | 1.55 | | 2.22 | |
| Q6ZD29 | Os08g0374000 protein | 1.24 | 1.04 | | 1.37 | |
| A0A0P0WB96 | Os04g0474800 protein (Fragment) | 1.23 | 1.17 | | 0.61 | |
| Q8GS72 | Os07g0640100 protein | 1.23 | 1.03 | | 1.53 | |
| Q0E1I1 | Os02g0439700 protein | 1.23 | 1.18 | | 1.79 | |
| Q6ZBX9 | Os08g0562600 protein | 1.22 | 1.67 | | 2.61 | |
| Q6K623 | Os02g0612900 protein | 1.22 | 1.39 | | 2.28 | |
| Q84SC3 | Os08g0162800 protein | 1.22 | 1.15 | | 1.80 | |
| Q67VC7 | Os06g0237300 protein | 1.21 | 1.09 | | 1.68 | |
| Q7XW32 | OSJNBb0062H02.10 protein | 1.19 | 1.08 | | 1.05 | |
| C7J6Y0 | Os09g0482780 protein | 1.18 | 0.91 | | 1.69 | |
| Q6Z674 | Os02g0720600 protein | 1.18 | 1.05 | | 1.48 | |
| Q5Z6B8 | Os06g0530200 protein | 1.17 | 0.99 | | 1.62 | |
| Q5JML5 | Os01g0754500 protein | 1.17 | 1.03 | | 1.57 | |
| Q9FP25 | Os01g0303000 protein | 1.16 | 1.87 | | 3.92 | |
| A0A0N7KMW0 | Os07g0129200 protein (Fragment) | 1.16 | 1.38 | | 1.84 | |
| B9G4B3 | Os09g0491772 protein | 1.15 | 1.29 | | 2.07 | |
| Q6AUG4 | Os05g0563550 protein | 1.14 | 1.02 | | 1.62 | |
| A0A0P0WI62 | Os05g0149300 protein (Fragment) | 1.13 | 1.07 | | 1.54 | |
| Q9FW35 | Os05g0101200 protein | 1.13 | 1.18 | | 1.55 | |
| Q8LJ05 | Os01g0698000 protein | 1.10 | 1.20 | | 1.81 | |
| A3BYP4 | Os09g0407700 protein | 1.09 | 1.12 | | 2.01 | |
| B7FAF1 | Os03g0222600 protein | 1.09 | 1.02 | | 1.70 | |
| Q6ZH84 | Os02g0593700 protein | 1.09 | 2.08 | | 2.26 | |
| A0A0P0WMW0 | Os05g0432700 protein (Fragment) | 1.09 | 1.28 | | 2.12 | |
| Q942Z3 | Os01g0934100 protein | 1.08 | 1.05 | | 2.59 | |
| A0A0N7KG02 | Os02g0720900 protein (Fragment) | 1.08 | 0.97 | | 1.08 | |
| B9FCM4 | Os04g0626400 protein | 1.07 | 1.10 | | 1.70 | |
| Q69TW4 | Os06g0211300 protein | 1.07 | 1.07 | | 1.72 | |
| A0A0P0XXQ7 | Os10g0568900 protein (Fragment) | 1.07 | 1.00 | | 1.52 | |
| Q6K700 | Os02g0821900 protein | 1.06 | 1.58 | | 1.66 | |
| A0A0P0XRR7 | Os09g0568900 protein (Fragment) | 1.06 | 0.89 | | 2.21 | |
| Q2RAK8 | Os11g0147800 protein | 1.06 | 0.56 | | 0.30 | |
| Q8H5M0 | Os07g0585000 protein | 1.06 | 1.10 | | 1.73 | |
| Q2QNI4 | Os12g0564400 protein | 1.03 | 0.89 | | 1.56 | |
| Q5VPH3 | Os06g0137600 protein | 1.03 | 0.94 | | 0.48 | |
| A0A0P0VQT9 | Os02g0793700 protein (Fragment) | 1.02 | 1.23 | | 1.28 | |
| C7J4C3 | Os06g0704200 protein (Fragment) | 1.01 | 1.05 | | 0.66 | |
| Q6F385 | Expressed protein | 1.01 | 1.60 | | 0.97 | |
| B7E707 | Os09g0509200 protein | 1.00 | 1.11 | | 1.65 | |
| Q6K5H8 | Os02g0600200 protein | 1.00 | 0.72 | | 0.51 | |
| Q8LMW8 | Os10g0191300 protein | 1.00 | 0.91 | | 0.65 | |
| Q8RZW7 | Os01g0916400 protein | 1.00 | 1.06 | | 1.53 | |
| Q6ETK1 | Os02g0180200 protein | 0.99 | 1.07 | | 1.30 | |
| Q5TKJ2 | Os05g0429400 protein | 0.99 | 0.85 | | 0.66 | |
| Q2QND6 | Os12g0569500 protein | 1.00 | 0.97 | | 1.69 | |
| Q6Z0W5 | Os02g0308400 protein | 0.97 | 0.89 | | 1.90 | |
| Q75T45 | Os12g0555000 protein | 0.94 | 1.39 | | 1.41 | |
| Q69XG4 | Os06g0610800 protein | 0.93 | 0.94 | | 0.94 | |
| A0A0P0YD24 | Os12g0640600 protein | 0.92 | 0.65 | | 0.74 | |
| Q0DJC0 | Os05g0302700 protein | 0.92 | 0.76 | | 0.51 | |
| Q75LD8 | Os03g0843400 protein | 0.91 | 1.25 | | 0.53 | |
| Q6ZLQ0 | Os07g0150100 protein | 0.90 | 0.93 | | 0.56 | |
| B7E914 | Os04g0310500 protein | 0.88 | 0.91 | | 2.99 | |
| Q8S3S1 | Os02g0708400 protein | 0.86 | 0.96 | | 0.54 | |
| Q0IZR7 | Os09g0553900 protein | 0.85 | 0.89 | | 0.66 | |
| Q6H713 | Os02g0170100 protein | 0.84 | 0.88 | | 1.53 | |
| Q0JCX3 | Os04g0445200 protein | 0.82 | 0.93 | | 0.38 | |
| Q84SC5 | Os08g0162600 protein | 0.82 | 0.88 | | 0.64 | |
| Q94CW5 | Os01g0862200 protein | 0.80 | 1.03 | | 0.76 | |
| Q8H4P7 | Os07g0147500 protein | 0.80 | 0.81 | | 0.44 | |
| Q0D8H0 | Os07g0160400 protein | 0.79 | 0.64 | | 0.90 | |
| Q2QWN3 | Os12g0189400 protein | 0.78 | 1.04 | | 0.49 | |
| Q5JK67 | Os01g0958100 protein | 0.77 | 0.74 | | 0.63 | |
| Q7EYM8 | Os08g0379400 protein | 0.76 | 0.85 | | 0.81 | |
| Q7XVP0 | OSJNBa0023J03.8 protein | 0.79 | 0.98 | | 1.05 | |
| Q0JBE3 | Os04g0538100 protein (Fragment) | 0.76 | 0.82 | | 0.64 | |
| A0A0P0X2H7 | Os07g0158300 protein (Fragment) | 0.76 | 0.82 | | 0.66 | |
| Q60E66 | Os05g0387200 protein | 0.75 | 1.03 | | 0.64 | |
| Q0D8N4 | Os07g0143000 protein | 0.75 | 0.67 | | 0.84 | |
| A0A0P0XWA7 | Os10g0492000 protein (Fragment) | 0.74 | 0.84 | | 0.79 | |
| Q0D6L9 | Os07g0467200 protein | 0.73 | 1.64 | | 1.23 | |
| Q0JAF4 | Os04g0602100 protein | 0.73 | 0.82 | | 0.99 | |
| Q7XUT4 | OSJNBa0042L16.6 protein | 0.73 | 0.74 | | 0.58 | |
| Q10LV7 | Os03g0333400 protein | 0.72 | 1.03 | | 1.25 | |
| Q0JFE2 | Os04g0111200 protein (Fragment) | 0.72 | 0.80 | | 0.79 | |
| A0A0N7KKC7 | Os05g0218400 protein | 0.72 | 0.97 | | 0.51 | |
| Q6ZL61 | Os07g0182100 protein | 0.72 | 0.98 | | 2.40 | |
| Q943K1 | Os01g0869800 protein | 0.71 | 0.67 | | 0.39 | |
| Q943W1 | Os01g0501800 protein | 0.71 | 0.87 | | 0.92 | |
| Q6YW78 | Os08g0382400 protein | 0.71 | 0.80 | | 1.14 | |
| Q6ZFH9 | Os08g0503200 protein | 0.71 | 0.89 | | 0.60 | |
| Q8S7H8 | Os03g0778100 protein | 0.71 | 0.76 | | 0.25 | |
| Q6ATY4 | UPF0603 protein Os05g0401100, chloroplastic | 0.70 | 0.80 | | 0.58 | |
| A0A0P0XMX9 | Os09g0453800 protein | 0.70 | 0.78 | | 0.66 | |
| A0A0N7KG12 | Os02g0729400 protein (Fragment) | 0.70 | 0.74 | | 0.65 | |
| A0A0P0X334 | Os07g0176900 protein (Fragment) | 0.69 | 0.90 | | 0.87 | |
| Q6ZBV1 | Os08g0200300 protein | 0.69 | 0.64 | | 0.40 | |
| Q0DJF9 | Os05g0291700 protein | 0.69 | 0.86 | | 0.76 | |
| Q0E032 | Os02g0581100 protein | 0.69 | 0.84 | | 0.09 | |
| A0A0P0XNM8 | Os09g0481200 protein (Fragment) | 0.69 | 0.92 | | 0.64 | |
| Q6ZGM0 | Os02g0117100 protein | 0.69 | 0.92 | | 0.84 | |
| Q69UU3 | Os07g0108300 protein | 0.68 | 0.97 | | 0.70 | |
| Q60EY9 | Os05g0208000 protein | 0.68 | 0.57 | | 0.50 | |
| Q2QSR7 | Os12g0420200 protein | 0.67 | 0.95 | | 0.54 | |
| Q0DEU8 | Os06g0133800 protein (Fragment) | 0.67 | 0.89 | | 0.63 | |
| A0A0P0WYZ5 | Os06g0611900 protein (Fragment) | 0.67 | 0.91 | | 0.66 | |
| Q0IWS0 | Os10g0492300 protein | 0.66 | 0.98 | | 0.55 | |
| A0A0P0XF80 | Os08g0347500 protein | 0.66 | 0.94 | | 1.02 | |
| Q652L5 | Os09g0567350 protein | 0.66 | 0.91 | | 0.42 | |
| Q0JEB0 | Os04g0303900 protein | 0.66 | 0.90 | | 1.17 | |
| Q7F9Y6 | OSJNBa0086O06.22 protein | 0.66 | 1.00 | | 0.74 | |
| Q0J3S0 | Os08g0557100 protein (Fragment) | 0.66 | 0.73 | | 0.59 | |
| Q5VP52 | Os06g0145800 protein | 0.66 | 0.79 | | 0.72 | |
| Q69NF7 | Os09g0530000 protein | 0.65 | 0.92 | | 0.49 | |
| Q69WH2 | Os06g0332800 protein | 0.65 | 1.03 | | 2.43 | |
| Q7X7H3 | OSJNBa0076N16.12 protein | 0.65 | 0.90 | | 0.58 | |
| Q69LC0 | Os07g0171100 protein | 0.65 | 0.85 | | 0.71 | |
| A0A0P0WXE9 | Os06g0531200 protein (Fragment) | 0.64 | 0.79 | | 0.46 | |
| Q84NW1 | Os07g0513000 protein | 0.64 | 0.83 | | 0.51 | |
| Q6H444 | Os09g0279400 protein | 0.64 | 0.68 | | 0.63 | |
| Q9LWT6 | Os06g0114000 protein | 0.63 | 0.82 | | 0.73 | |
| Q8L472 | Os07g0105600 protein | 0.63 | 1.07 | | 1.10 | |
| Q2QMC5 | Os12g0609500 protein | 0.62 | 0.91 | | 0.75 | |
| Q6Z8N7 | Os08g0512500 protein | 0.62 | 0.93 | | 0.85 | |
| Q5Z8V3 | Os06g0705100 protein | 0.60 | 0.87 | | 0.95 | |
| Q69UI8 | Os08g0116500 protein | 0.59 | 1.03 | | 0.77 | |
| Q53N80 | Os11g0242400 protein | 0.59 | 0.87 | | 0.50 | |
| Q6YU90 | Os02g0101500 protein | 0.59 | 0.94 | | 0.59 | |
| Q5Z6P4 | Os06g0264800 protein | 0.58 | 0.87 | | 0.34 | |
| A0A0P0V7A3 | Os01g0711400 protein (Fragment) | 0.58 | 0.79 | | 0.80 | |
| B9FM04 | Os05g0104650 protein | 0.58 | 0.71 | | 0.55 | |
| Q6YZK5 | Os08g0359000 protein | 0.58 | 0.51 | | 0.44 | |
| A0A0P0WN55 | Os05g0456300 protein (Fragment) | 0.53 | 0.90 | | 0.83 | |
| A0A0P0VQ66 | Os02g0773300 protein | 0.51 | 0.80 | | 0.69 | |
| Q10N30 | Os03g0284400 protein | 0.50 | 0.83 | | 2.03 | |
| Q9ZWF5 | Os05g0568300 protein | 0.44 | 0.92 | | 0.67 | |
| A0A0P0W2S8 | Os03g0704100 protein (Fragment) | 0.37 | 0.58 | | 0.42 | |

a: the protein ID come from UniProt databse.

b: T0 represents the control group without cold treatment; T1 represents cold treatment for 24 h; T2 represents cold treatment for 72 h; T3 represents cold treatment for 120 h;
